# Structural and functional analyses of *Burkholderia pseudomallei* BPSL1038 reveal a Cas-2/VapD nuclease sub-family

**DOI:** 10.1038/s42003-023-05265-4

**Published:** 2023-09-08

**Authors:** Sofiyah Shaibullah, Nurshahirah Shuhaimi, De-Sheng Ker, Nurhikmah Mohd-Sharif, Kok Lian Ho, Aik-Hong Teh, Jitka Waterman, Thean-Hock Tang, Rui-Rui Wong, Sheila Nathan, Rahmah Mohamed, Min Jia Ng, Shin-Yee Fung, Mohd Anuar Jonet, Mohd Firdaus-Raih, Chyan Leong Ng

**Affiliations:** 1 Institute of Systems Biology, Universiti Kebangsaan Malaysia, UKM Bangi, 43600 Selangor Malaysia; 2 Department of Pathology, Faculty of Medicine and Health Sciences, Universiti Putra Malaysia, UPM Serdang, 43400 Selangor Malaysia; 3 Centre for Chemical Biology, Universiti Sains Malaysia, Bayan Lepas, 11900 Penang Malaysia; 4 Diamond Light Source, Harwell Science & Innovation Campus, Didcot, Oxfordshire OX11 0DE UK; 5 Advanced Medical and Dental Institute, Universiti Sains Malaysia, Pulau Pinang, Malaysia; 6 Department of Biological Sciences and Biotechnology, Faculty of Science and Technology, Universiti Kebangsaan Malaysia, UKM Bangi, 43600 Selangor Malaysia; 7 Medicinal Mushroom Research Group (MMRG), Department of Molecular Medicine, Faculty of Medicine, University of Malaya, Kuala Lumpur, Malaysia; 8 Malaysia Genome and Vaccine Institute, National Institutes of Biotechnology Malaysia (NIBM), Jalan Bangi, Kajang, 43000 Selangor Malaysia; 9 Department of Applied Physics, Faculty of Science and Technology, Universiti Kebangsaan Malaysia, UKM Bangi, 43600 Selangor Malaysia; 10 Present Address: Department of Biochemistry, University of Cambridge, Cambridge, CB2 1GA UK; 11 Present Address: Faculty of Health and Life Sciences, Inti International University, Persiaran Perdana, BBN, Nilai, 71800 Negeri Sembilan Malaysia

**Keywords:** X-ray crystallography, Proteins

## Abstract

*Burkholderia pseudomallei* is a highly versatile pathogen with ~25% of its genome annotated to encode hypothetical proteins. One such hypothetical protein, BPSL1038, is conserved across seven bacterial genera and 654 *Burkholderia* spp. Here, we present a 1.55 Å resolution crystal structure of BPSL1038. The overall structure folded into a modified βαββαβα ferredoxin fold similar to known Cas2 nucleases. The Cas2 equivalent catalytic aspartate (D11) pairs are conserved in BPSL1038 although *B. pseudomallei* has no known CRISPR associated system. Functional analysis revealed that BPSL1038 is a nuclease with endonuclease activity towards double-stranded DNA. The DNase activity is divalent ion independent and optimum at pH 6. The concentration of monovalent ions (Na^+^ and K^+^) is crucial for nuclease activity. An active site with a unique D^11^(X20)SST motif was identified and proposed for BPSL1038 and its orthologs. Structure modelling indicates the catalytic role of the D^11^(X20)SST motif and that the arginine residues R10 and R30 may interact with the nucleic acid backbone. The structural similarity of BPSL1038 to Cas2 proteins suggests that BPSL1038 may represent a sub-family of nucleases that share a common ancestor with Cas2.

## Abstract (web summary)

A crystal structure of the *Burkholderia pseudomallei* protein, BPSL1038, and subsequent functional analyses suggests that it acts as an endonuclease and may represent a subfamily of nucleases that share a common ancestor with Cas2.

## Introduction

*Burkholderia pseudomallei* (BP) is a pathogenic Gram-negative soil bacterium that has been classified as a Tier 1 select agent due to its biothreat potential for causing melioidosis, a disease with high fatality rates in humans and animals^1^. The bacterium is also highly resistant to clinically used antibiotics^2^. The genome size of *B. pseudomallei* strain K96243, an often used reference genome, is 7.24 Mbp and contains 5855 coding DNA sequences (CDSs), with approximately 25% of the genome annotated to encode for hypothetical proteins^3,4^.

The large number of genes encoding for proteins with unknown functions have posed a challenge in fully elucidating the details related to melioidosis pathogenesis to understand the bacterium’s capacity to survive in various hostile environments and host organisms^5,6^. Many *B. pseudomallei* hypothetical proteins are conserved throughout the *Burkholderiaceae* family.

One such protein, BPSL1038, is found in at least 654 members of the family with available genomes (https://www.burkholderia.com/orthologs/list?id=371057)^4^. This number accounts for species that are known pathogens as well as those that are not known to be pathogenic. In the GenBank non-redundant protein sequence (nr) database (27^th^ April 2023), 249 orthologous sequences of BPSL1038 in the *Burkholderiaceae* family from the genera *Burkholderia*, *Paraburkholderia*, *Caballeronia*, *Trinickia, Robbsia, Pararobbsia*, *Mycetohabitans* and *Mycoavidus* were retrievable. In addition to the *Burkholderiaceae* family, homologs from *Pseudomonas* and *Bacillus* are also in the database, indicating that this protein is prevalent in nature and not unique to only the *Burkholderiaceae* family (Supplementary Fig. 1). The highly conserved nature of BPSL1038 in *Burkholderiaceae* and its presence in other bacterial families, despite having no detectable sequence orthologs of known functions, prompted us to investigate its function using a structure-guided characterization approach.

Here, we report the crystal structure of BPSL1038 that shares high structural similarities to Cas2 (CRISPR-associated protein 2) and VapD (virulence-associated protein D) proteins. Functional assays demonstrated that BPSL1038 has nuclease activity. Our structural analysis was able to identify an active site and from there allowed us to propose a potential mechanism of how the protein interacts with and cleaves its nucleic acid substrate.

## Results

### Overall structure of BPSL1038

**Fig. 1:**
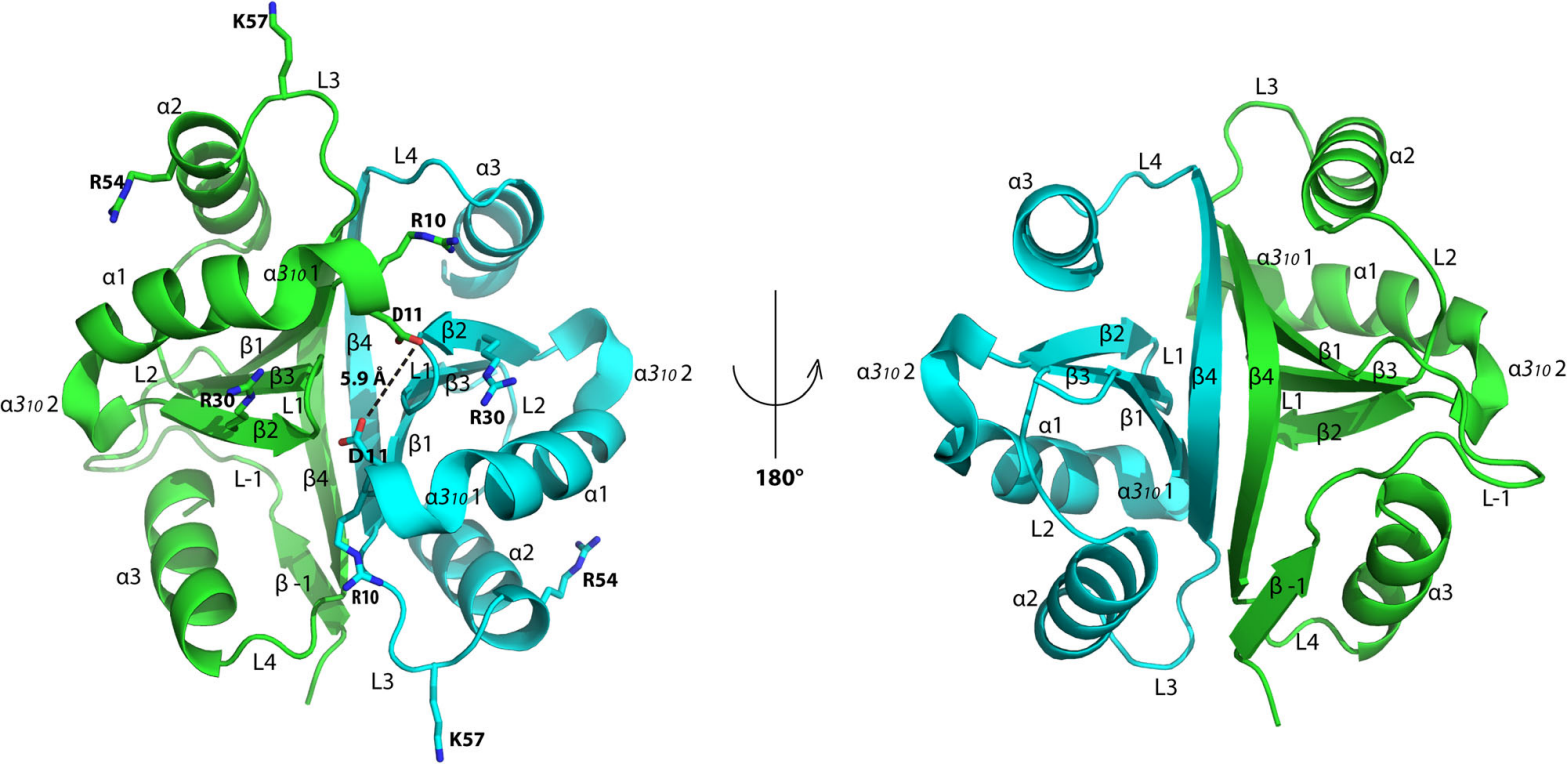
Crystal structure of homodimeric BPSL1038 and electrostatic potential compared to Cas2 homologs. Both protomers A (Green) and B (Cyan) adopt a βαββαβ ferredoxin fold with an appended C-terminal helix. The proposed catalytic active aspartate pair of D11 is 5.9 Å apart.

The crystal structure of recombinant BPSL1038 (rBPSL1038) protein (~12 kDa) was determined by the single wavelength anomalous (SAD) dispersion method using the SeMet-BPSL1038 protein. The SeMet-BPSL1038 (smBPSL0138) and native BPSL1038 (rBPSL1038) crystals diffracted to 1.88 Å and 1.55 Å resolution, respectively. Both crystals belong to space group C222_1_ with similar unit cells (Table 1). The rBPSL1038 crystals contain two protein molecules per asymmetric unit forming a symmetric homodimer. The homodimeric structure of smBPSL0138 and rBPSL1038 show that both structures are almost identical with a root mean square deviation (r.m.s.d) of 0.15 Å over 187 Cα atoms (Supplementary Fig. 2a). The structure of each rBPSL1038 protomer adopted a modified βαββαβα ferredoxin fold (Fig. 1). The α1 helix (residues 1–72) is flanked by two 3_10_-helices while the α3 helix is appended at the C-terminal region (residues 76–86). The long β4 strand (residues 61–71) contains a kink at residue T67 that results in a sharp curved shape. One end of the β4 strand (residues 61–67) from one protomer forms an anti-parallel β-sheet with the end of the β4 strand (residues 68–71) of the other protomer, assembling the dimerization backbone of the BPSL1038 dimer. The β4 strand further forms a five-stranded antiparallel β-sheet together with four β-strands from the other protomer. Overall, protomer A has a well-ordered fusion 6xHis-tag (13 amino acids) at the N-terminal region with residues -11 to -8 forming an additional β1 strand (Fig. 1). The β1 strand forms an anti-parallel β-sheet with strand β4. Both subunits (residues 1–87) are highly similar with an r.m.s.d of 1.3 Å over 87 Cα atoms (residues 1–87); the biggest deviation was observed at loop L4 and helix α3 regions and is likely due to the existence of the fusion His-tag β1 strand that interacts with the β4 strand of protomer A (Supplementary Fig. 2b). The SEC analysis of rBPSL1038 with and without the N-terminus 6X-His fusion tag was found to be very similar, suggesting that the BPSL1038 dimer is stable without the ordered β1-β4 anti-parallel β-sheet in protomer A that was observed in the crystal structure (Supplementary Fig. 3).

**Table 1:** Crystallographic statistics for selenomethionine labelled and native protein crystals of BPSL1038.

	Seleno methionine	Native	Native (soaked with 25 mM manganese)
Wavelength (Å)	0.97972	0.97950	1.54187
Resolution range (Å)	28.97–1.88 (1.92 –1.88)	28.08–1.55 (1.58–1.55)	47.73–2.05 (2.11–2.05)
Space group	C222_1_	C222_1_	C222_1_
Unit cell			
a, b, c (Å)	85.73, 115.89, 46.81	85.36, 115.63, 46.73	85.46, 115.54, 46.79
α, β, γ (˚)	90.0, 90.0, 90.0	90.0, 90.0, 90.0	90.0, 90.0, 90.0
Measured reflections	123762	258112	90950
Unique reflections	19376	33712	14932
R_sym_	0.096 (0.645)	0.084 (0.563)	0.130 (0.556)
R_pim_	0.041 (0.312)	0.031 (0.240)	0.056 (0.312)
CC (1/2)	0.997 (0.826)	0.996 (0.896)	0.991 (0.810)
Mean I/σI (I)	13.4 (2.4)	12.3 (2.9)	9.7 (2.3)
Multiplicity	6.4 (5.0)	7.7 (5.3)	6.1 (4.1)
Mosaicity (˚)	0.09	0.30	0.57
Completeness (%)	99.6 (94.6)	99.1 (89.8)	99.9 (99.6)
Refinement statistics			
R_cryst_, R_free_ (%)	14.3, 17.5	11.8, 15.7	21.8, 25.8
No. of molecules per asymmetric unit	2	2	2
No. of water molecules	158	161	213
No. of beta-mercaptoethanol molecules	6	-	-
No. of Sodium ions	2	1	1
Root mean square deviation from ideal values (r.m.s.d.)			
Bond length (Å)	0.019	0.022	0.016
Bond angle (°)	2.10	2.05	1.715
Ramachandran plot statistics			
Favoured (%)	94.8	95.3	98.5
Allowed regions (%)	5.2	4.7	
Outliers (%)	0	0	1.1
Average B factors (All atoms)	33.9	29.4	21.48
Deposited structure factors and coordinates	PDB Id: 7VXT	PDB Id: 7VXR	Supplementary Data 2–3

In addition to the anti-parallel β4 sheet, the BPSL1038 dimer interface includes β1 to η1 (residues 6–11), β2 to β3 (residues 31–37), α2 helix (residues 44, 45, 48 and 52), α2-β4 loop, and α3 helix with a total calculated buried interface area of 1494 Å^2^. This accounts for ~25% of the protomer surface area and involves mainly hydrophobic interactions in addition to 16 hydrogen bonds and one salt bridge. In total, 42% (37 residues) of the total amino acid content is associated with dimerization. A Complex Formation Significance Score (CSS) of 0.855 from PISA interface analysis indicates that BPSL1038 is a stable dimer, that agrees with our previous report^7^.

### Analysis of structure homologs

A DALI server search^8,9^ identified proteins or domains with ferredoxin-like folds as having detectable and significant fold similarity to the BPSL1038 protomer despite having very low sequence identity (5–16%). Among the ferredoxin-like folds retrieved by DALI are the domain III of elongation factor 2 from *Candidatus Methanoperedens nitroreducens* and *Pyrococcus horikoshii* (Z-score 7.6, PDB ID 6U45 and 5H71 r.m.s.d ~3.0 Å), the domain III of ribosome maturation protein SDOI homolog (Z-score 7.5, PDB ID: 2WBM r.m.s.d 3.5 Å), LepA (Z-score 7.3, PDB ID 3CB4 r.m.s.d 2.6 Å) and several CRISPR-associated Cas2 proteins from *Thermococcus onnurineus* (Z-score 7.05, PDB ID: 5G4D r.m.s.d 3.0 Å), *Pyrococcus furiosus* DSM 3638 (Z-score ~7.5, PDB ID: 4TNO and 2I0X r.m.s.d ~ 3.0 Å) and *Thermus thermophilus* (Z-score 7.3, PDB ID: 1ZPW r.m.s.d 2.9 Å).

Further pairwise structure comparisons of the results retrieved by DALI found that the overall homodimeric folding of BPSL1038 is very similar to those of homodimeric CRISPR-associated Cas2 proteins from *Pyrococcus furiosus* (PDB IDs: 4TNO and 2I0X, r.m.s.d of 2.9 Å over 144 aligned Cα atoms); *Thermus thermophilus* (PDB ID: 1ZPW, 2.5 Å r.m.s.d over 142 aligned Cα atoms), *Thermococcus onnurineus* (PDB ID: 5G4D, r.m.s.d 2.9 Å over 135 aligned Cα atoms); and *Streptococcus pyogenes* serotype M1 (PDB ID: 4QR2, r.m.s.d 3.3 Å over 135 aligned Cα atoms) (Supplementary Fig. 4).

### Sequence and structure comparison of BPSL1038 and CRISPR-associated Cas2 proteins

**Fig. 2:**
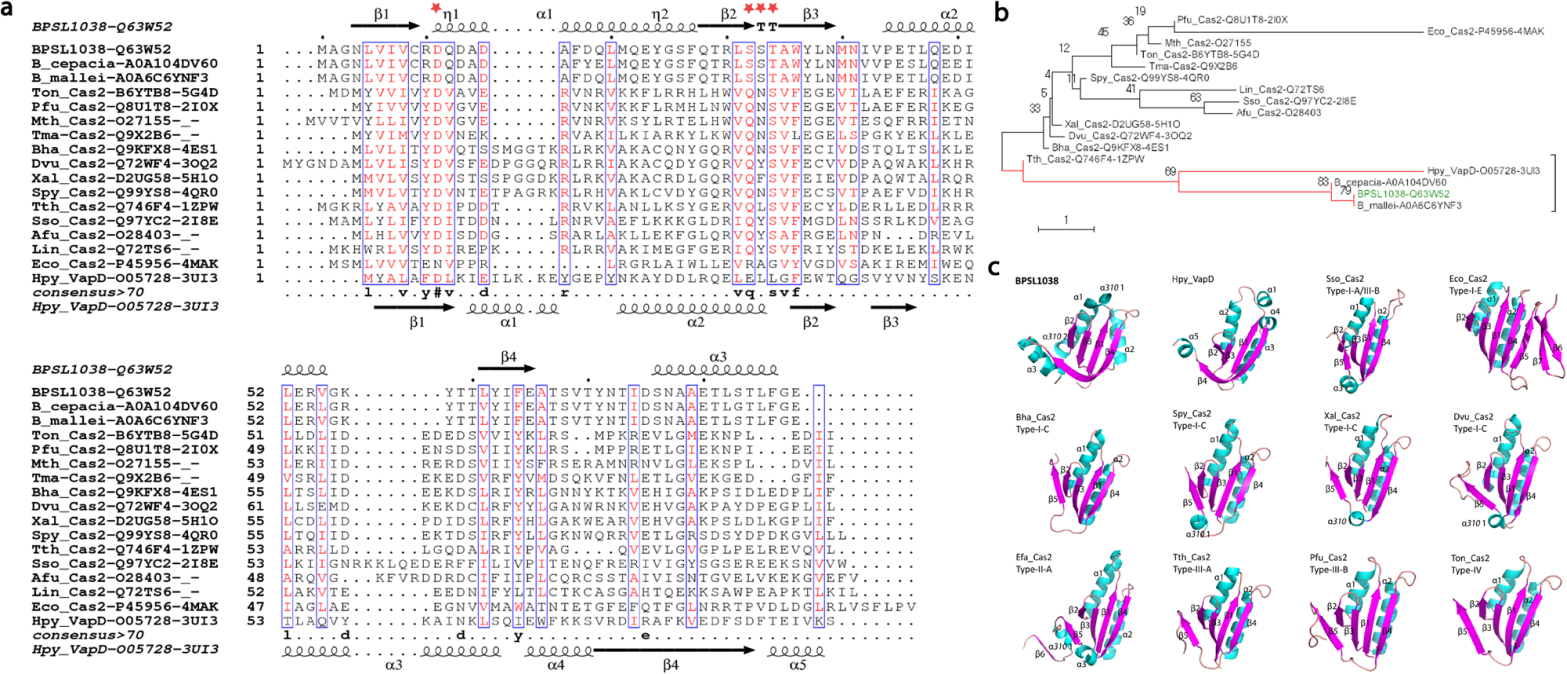
Sequence analysis of BPSL1038, CRISPR-associated Cas2 proteins and VapD protein. **a** Multiple sequence alignment of BPSL1038 with CRISPR-associated Cas2 proteins and VapD protein from archaea and bacteria that share high structural similarity but low sequence identity (10-15%). The D^11^'(X20)SST motif of BPSL1038 is indicated with stars in red. The proteins are named using the format Organism abbreviation_Cas2-UniProtKB ID-PDB ID. Afu: *Archaeoglobus fulgidus*, Bha: *Bacillus halodurans*, Dvu: *Desulfovibrio vulgaris*, Eco: *Escherichia coli*, Hpy: *Helicobacter pylori*, Lin: *Leptospira interrogans*, Mth: *Methanothermobacter thermautotrophicus*, Pfu: *Pyrococcus furiosus*, Spy: *Streptococcus pyogenes*, Sso: *Saccharolobus solfataricus*, Tma: *Thermotoga maritima*, Ton: *Thermococcus onnurineus*, Tth: *Thermus thermophilus*, Xal: *Xanthomonas albilineans*. VapD is VapD protein. The multiple sequence alignment was performed using CLUSTAL W^59^ and the secondary structure was incorporated with ESPript 3.0. **b** The multiple sequence alignment results were used to obtain the phylogenetic relationship of BPSL1038 with CRISPR-associated Cas2 proteins and VapD protein using Neighbor-Joining^60^ within MEGA7^61^. The optimal tree with the sum of branch length 24.97425156 is shown. The percentage of replicate trees in which the associated taxa clustered together in the bootstrap test (1000 replicates) are shown next to the branches^62^. The tree is drawn to scale, with branch lengths in the same units as those of the evolutionary distances used to infer the phylogenetic tree. The evolutionary distances were computed using the Poisson correction method^63^ and are in the units of the number of amino acid substitutions per site. The analysis involved 15 amino acid sequences. All ambiguous positions were removed for each sequence pair. **c** Structure comparison of BPSL1038 and Cas2 homologs from four types of CRISPR-Cas systems. All the protomers are shown with cyan helices and magenta β-strands. All the homologs were superimposed on BPSL1038 and are shown in similar orientation. *Helicobacter pylori* 26695 VapD (PDB ID: 3IU3), Sso_Cas2: *Saccharolobus solfataricus* P2 Cas2 (PDB ID: 2I8E), Eco_Cas2: *Escherichia coli* K-12 Cas2 (PDB ID:5DQT), Bha_Cas2*: Bacillus halodurans* C-125 Cas2 (PDB ID: 4ES1), Spy_Cas2: *Streptococcus pyogenes* serotype M1 Cas2 (PDB ID: 4QR2), Xal_Cas2: *Xanthomonas albilineans GPE PC73* Cas2 (PDB ID:5H1P), Dvu_Cas2: *Desulfovibrio vulgaris* str. Hildenborough Cas2 (PDB ID: 3OQ2), Efa_Cas2: *Enterococcus faecalis* TX0027 (PDB ID: 5XVN), Tth_Cas2: *Thermus thermophilus* (PDB id: 1ZPW), Pfu_Cas2: *Pyrococcus furiosus* DSM 3638 Cas2 (PDB Id: 4TNO) and Ton_Cas2: *Thermococcus Onnurineus* Cas2 (PDB ID: 5G4D).

Multiple sequence alignment using CLUSTAL W showed that despite low sequence similarity between BPSL1038 and Cas2 proteins retrieved by DALI (10–15%), several conserved residues in Cas2 are aligned (Fig. 2a). Most of these residues (I7, V8, L20, L31, S32, T34, A35, W36, L52, V55, L61, F64) are structurally important for either dimerization or intramolecular hydrophobic interaction within a protomer. D11 is highly conserved among the Cas2 proteins and proposed to form a residue pair with the D11 side chain from a different dimer chain, which together, chelate specific divalent metal ions which are important for nuclease activities. The phylogenetic relationship analysis of BPSL1038 with CRISPR-associated Cas2 proteins and VapD protein using Neighbour-Joining indicates that BPSL1038 is more similar to the VapD protein from *Helicobacter pylori* (Hpy_VapD) than other CRISPR-Cas2 associated proteins (Fig. 2b).

**Fig. 3:**
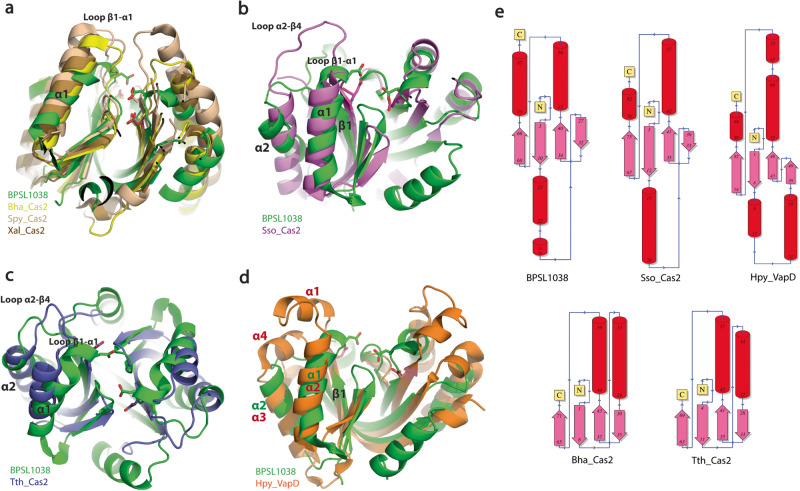
Structure comparison of BPSL1038 and CRISPR Cas2-associated proteins. **a** Superimposition of BPSL1038 with Bha_Cas2, Spy_Cas2 and Xal_Cas2 with known DNase activity that cleaves double-stranded DNA (dsDNA). **b** Superimposition of BPSL1038 with Sso_Cas2 that is known to cleave single-stranded RNA (ssRNA). **c** Superimposition of BPSL1038 with Tth_Cas2 that is known to cleave dsDNA and ssRNA. **d** Superimposition of BPSL1038 with Hpy_VapD that is known to cleave mRNA. The β1-α1 and α2-β4 loops that were proposed as DNA and RNA recognition loops, respectively, are shorter in BPSL1038. Hpy_VapD has a helix formed in those respective loop regions which are not found in BPSL1038 and other Cas2 proteins. The catalytic active aspartate residue pairs are shown in stick format. **e** 2D topology diagram of BPSL1038, Bha_Cas2, Sso_Cas2, Tth_Cas2 and Hpy_VapS monomer generated using PDBsum^64^.

Overall structure comparisons of BPSL1038 with Cas2 and Hpy_VapD proteins revealed substantial structural differences at α1, loop β1-α1, loop α2-β4, β4 and the C-terminal regions although all structures adopted the conserved βαββαβ ferredoxin fold (Fig. 2c). In BPSL1038, α-3_10_1 accommodates the active site residue D11, while the equivalent residue in Cas2 protein is generally located at the β1 region (Figs. 1, 3). As a result, BPSL1038 has a short and rigid β1-α1 turn compared to Cas2 proteins such as Bha_Cas2, Spy_Cas2 and Xal_Cas2 that are known to cleave dsDNA^10–12^ (Fig. 3). The larger and flexible β1-α1 loop was proposed as a DNA binding feature of Cas2 DNase as it can form a wider and shallower DNA binding groove^10^. BPSL1038 has a shorter α2-β4 loop, similar to known endoribonucleases such as Sso-Cas2 that are able to cleave ssRNA^13^. The long α2-β4 loop of Cas2 proteins was proposed as an RNA substrate recognition feature because it forms a deeper and narrower substrate binding groove that could accommodate ssRNA binding^14^. Nonetheless, Tth_Cas2 and several Cas2 proteins (Lin_Cas2, Afu_Cas2, Tma_Cas2 and Mth_Cas2) predicted to have a shorter α2-β4 loop also have RNase activity^15^. Hpy_VapD has a helix in this region and is able to cleave mRNA by purine-specific endoribonuclease activity^16^ (Fig. 3). Another significant structural difference between BPSL1038 and the Cas2 proteins is that BPSL1038 has a long β4 strand with a kink in the middle, thus making it more like Hpy_VapD (Fig. 2c). A similar region in the homologs of Cas2 generally has either a 3_10_-helix (Sso_Cas2, Spy_Cas2, Xal_Cas2 and Dvu_Cas2), an unstructured loop after a rather short β4 strand (Bha_Cas2, Tth_Cas2, Ton_Cas2 and Pfu_Cas2) or a β hairpin (Eco_Cas2) (Fig. 2c). The substrate induced conformational changes or motion of the β4-β5 hinge have been suggested to be important for the catalytic activation of Cas2 nucleases^10–13,15,17^.

The C-terminus of BPSL1038 is appended to an long α3 helix that is absent in currently available Cas2 protein structures. The Hpy_VapD structure has a short helix at the C-terminus (Figs. 2c, 3e). The C-terminal β strand of Cas2 proteins is important for Cas1-Cas2 complex formation^17–20^. This suggests that BPSL1038 may adopt a different mechanism in complex formation if it is capable of forming a Cas1-Cas2-like complex. Although both BPSL1038 and Hpy_VapD share similar structural features at the β4 strand and the C-terminus, BPSL1038 lacks the helices in the middle of loop β1-α1 and loop α2-β4 that are present in Hpy_VapD (Figs. 2c, 3e).

The crystal structure of Tth_Cas2 (Fig. 2c) and the predicted structure of Lin_Cas2 both have the short β1-α1 and α2-β4 loops; however, these proteins are able to cleave both DNA and RNA substrates^10,15^. Conversely, Cas2 proteins such as Dvu_Cas2 with long β1-α1 and α2-β4 loops and Ton_Cas2 with short β1-α1 and α2-β4 loops have no reported nuclease activity^21,22^, suggesting that Cas2 proteins can adopt a dynamic substrate-binding mode.

Structural divergence among Cas2 proteins has been reported to play a crucial role in nuclease activity and substrate specificity. In this context, the BPSL1038 structure was shown to have a modified α1 helix with an extended 3_10_ helix at loop β1-α1 and a short α2-β4 loop suggesting that the signature structural features for DNA or RNA binding as shown in some Cas2 nucleases are not conserved in BPSL1038. Furthermore, the extended kinked β4 strand of BPSL1038 results in it lacking the dynamic β4-β5 hinge region which previously described as playing an important role in regulating the activation of nuclease catalytic activity^11–13,17,21,22^, thus pointing to the possibility that BPSL1038 may employ a different catalytic activation mechanism.

### Catalytic active site and nucleic acid binding analysis

Despite the low sequence similarity and substantial structural divergence between BPSL1038 and Cas2 proteins, the conserved residue D11 at 3_10_ α1 in BPSL1038 is coordinated closely to the equivalent aspartate residues at the β1 strand of other Cas2 and VapD proteins that are central to catalysis (Figs. 1, 3). The aspartate pair has been proposed to coordinate with one divalent cation (Mg^2+^ or Mn^2+^) that are required to cleave phosphodiester bonds of DNA and RNA substrates^10–13,15^. In order to chelate the divalent cation, the distance between these two aspartate residues was proposed to be a crucial determinant of catalytic activity, where if they are too far apart, it would not be possible to chelate the metal ion^10^. To date, all existing Cas2 and VapD structures are suggested to be in the catalytically inactive conformation due to the aspartate pair being 6.5–15 Å apart, a distance that is unable to coordinate with the divalent metal ion^10,12^.

**Fig. 4:**
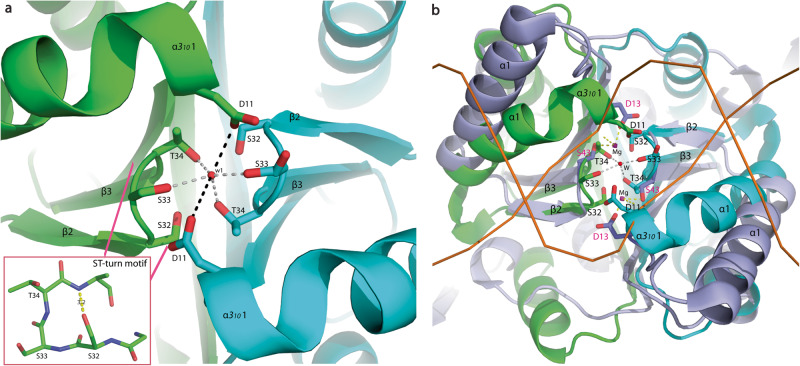
The putative catalytic active site of BPSL1038 dimer. **a** The D^11^(X20) SST motif forming a negatively charged cage coordinating a well-ordered water residue (w1). The distance between the D11 and w1 for subunit A and B are 3.2 Å and 3.6 Å, respectively. The typical ST-turn motif of BPSL1038 involved S32, S33 and T34 was boxed. **b** The SSM superposition of BPSL1038 with *Enterococcus faecalis* TX0027 Cas1-Cas2-prespacer DNA complex (PDB ID: 5XVN) (light blue) shows that the putative active site region of BPSL1038 shares high similarity to the D13 and S43 residues of Efa_Cas2 that coordinate with two Mg2+ ions (magenta) for prespacer DNA backbone contact^20^. The overall homodimeric structure of BPSL1038 is similar to the Efa_Cas2 with a r.m.s.d of 3.3 Å over 140 aligned Cα atoms.

In BPSL1038, the aspartate (D11) pair is 5.9 Å apart and located together with the S32, S33 and T34 residues at the β2-β3 turn that forms a negatively charged cage for coordinating a water molecule that is tetrahedrally hydrogen bonded to residues S33 and T34 from both subunits (Fig. 4). It may be possible for the coordination of the water molecule in BPSL1038 to be substituted by a structurally equivalent divalent metal ion within the limits of small conformational changes in the active site if BPSL1038 functions as a divalent metal ion-dependent nuclease. Interestingly, the β2-β3 turn forms an ST-turn motif with side chain oxygen atom of residue S32 hydrogen bonded to the main-chain NH of residue T34^23^. It is worth noting that the D^11^'(X20)SST potential nuclease active site motif is conserved across all aligned homologs in *Burkholderiacea*, *Pseudomonadaceae* and *Candidatus* species (Supplementary Fig. 1). A search for similar occurrences of the DSST structural motif in other PDB structures using the ASSAM computer program revealed a match to a member of the Ski-like DNA binding domain superfamily (PDB ID: 3EQ5). However, a similar search using the DSST-DSST arrangement from each protomer did not retrieve any similarly arranged motif to be present in the currently available PDB structures.

In the EfaCas1–2/prespacer complex (PDB ID: 5XVN), the D11^BPSL1038^ equivalent residue (D13) and S43^Efa_Cas2^ are associated with Mg^2+^ ions that interact with the minor groove of the prespacer DNA backbone (Fig. 4b)^20^, a similar observation was also found in the PAM prespacer bound Cas4/Cas1/Cas2 complex of *Geobacter sulfurreducens* (PDB:7MI4) with the equivalent D11^BPSL1038^ residue (D10) and S34^Gsu_Cas2^ associated Mn^2+^ ions^24^. Nonetheless, the crystal structure of rBPSL1038 soaked with manganese ions was found to be almost identical (RMSD of 0.165 Å) to the rBPSL1038 that was previously solved, in which a water molecule positioned in the active site was not to be substituted by a manganese ion. This is evident when a manganese atom was modelled into the manganese chloride soaked rBPSL1038 crystal structure; the refined structure shows that the Mn atom has a temperature factor (B-factor) value of 49. In contrast, the surrounding atoms only have a B-factor of ~10–20. In addition, negative density was found in the Fo-Fc difference map of the manganese ion. Nonetheless, when a water molecule was fitted to the density, the water molecule was refined with a B-factor of 13.2, similar to the B-factor of the surrounding atoms. These results indicate that the manganese atom did not replace the water molecule in the active site of BPSL1038 (Supplementary Fig. 5).

**Fig. 5:**
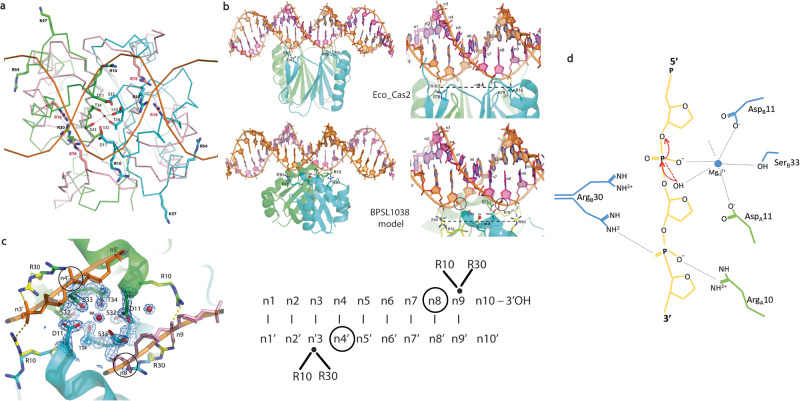
BPSL1038 may interact with double-stranded DNA via an arginine clamp feature similar to Eco_Cas2. **a** The SSM superposition of BPSL1038 dimer (green and cyan for subunit A and B, respectively) with the *E. coli* Cas2 protein dimer (Eco_Cas2, light pink) that interacts with DNA substrates in the Cas1-Cas2-protospacer DNA Complex (PDB ID: 5DS5). The overall homodimeric structure of BPSL1038 is similar to the Eco_Cas2 with r.m.s.d of 3.1 Å over 123 aligned Cα atoms. The R16 and R78 arginine clamp of Cas2 interacts with protospacer DNA. The residues R10 and R30 from each subunit of BPSL1038 share similar coordination with the ‘arginine clamp’ that may also interact with the DNA substrate. **b** The Eco_Cas2-DNA complex (PDB ID: 5DS5) is shown in the same orientation as BPSL1038-DNA model (for better comparison, Eco_Cas2 was coloured in green and cyan same as BPSL1038). The side chain rotamer conformation of BPSL1038 R10 and R30 is modelled (yellow) to interact with each strand of DNA. **c** A close-up view of the modelled dsDNA minor groove interacting with the proposed BPSL1038 ‘arginine clamp’. The D11 and SST active site of BPSL1038 is located between the ‘arginine clamp’ pair, which may accommodate one or two catalytic Mg^2+^ to cleave the DNA backbone before the arginine clamped nucleotide (circled) located close to the D11 aspartate pair (left). The 2Fo-Fc maps for the active site are contoured at 1.5σ. **d** Schematic presentation of the proposed DNA cleavage mechanism in which the aspartate pair coordinate with a metal cation (Mg^2+^) to activate a nucleophilic water which coordinated with the cation. The activated hydroxyl group of the water molecule will initiate an attack to the scissile phosphate. The negatively charged trigonal bipyramidal transition state is likely stabilized by the metal ion or the side chain of R30. The Mg^2+^ ion may also coordinate with residue S33.

Further structure analysis revealed that the rather flat and wide potential substrate-binding surface of BPSL1038 provides a suitable platform for dsDNA binding. The BPSL1038 dimer has an overall negatively charged surface that differs from other Cas2 examples (Supplementary Fig. 6). The side chains of four BPSL1038 solvent-exposed positively charged amino acids (R10, R30, R54 and K57) from each subunit are systematically oriented on the potential substrate binding surface, with the negatively charged cage formed by the D^11^SST motif located in the middle (Fig. 5). The structural arrangement of these four positively charged amino acids point to the possibility that they may have a direct role in nucleic acid binding. Using these positions as a reference point for a nucleic acid binding interface, we constructed a model that demonstrates how the putative active site of BPSL1038 is able to fit the minor groove space in double-stranded DNA spanning ~12 Å (see section on endonuclease activity). Superpositions of BPSL1038 with the *E. coli* Cas1-Cas2 complex bound to protospacer DNA substrates (PDB ID: 5DS5)^25^ indicates that the R10 and R30 residues of the BPSL1038 dimer may function as a pair of arginine clamps to interact with the phosphate backbone of the DNA substrate, similar to the R78 and R16 residues in Eco_Cas2. However, rotameric conformational changes of BPSL1038 R10 and R30 in the current coordination would be needed for such an interaction to occur (Fig. 5).

### Endonuclease activity of rBPSL1038

**Fig. 6:**
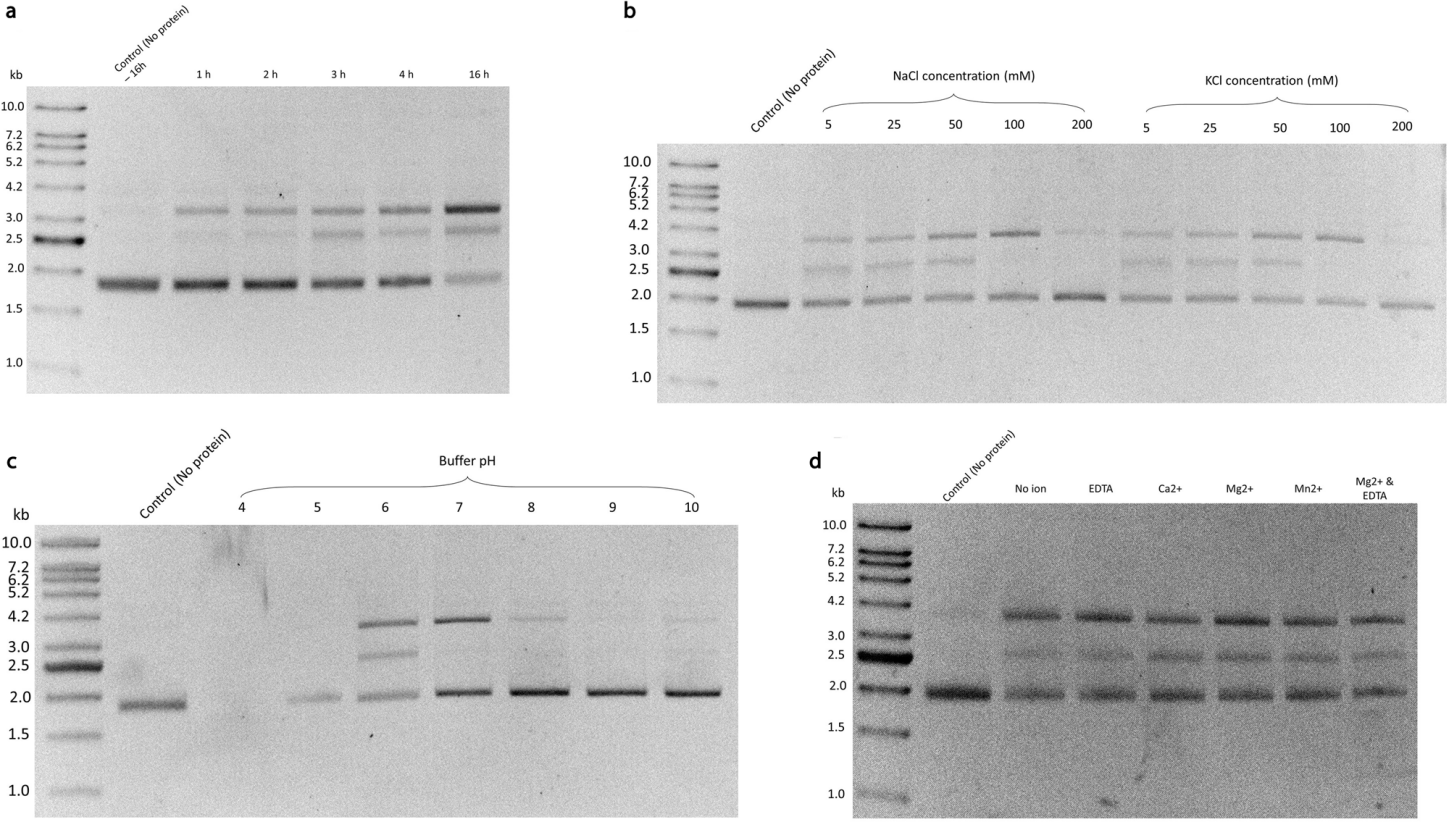
Nuclease activity of BPSL1038. **a** Incubation of pUC19 plasmid with rBPSL1038 shows the endonuclease sequential mechanism in nicking the plasmid by gradually transforming the supercoiled DNA to circular and linear forms. **b** Increasing concentrations (5, 25, 50, 100 and 200 mM) of monovalent salts (NaCl and KCl) showed gradual inhibition of rBPSL1038 nuclease activity. **c** Optimal nuclease activity were observed at pH 6, where at higher pH (pH 7 – 10) showed gradual inhibition, pH 4 showed precipitation. **d** A series of 2.5 mM divalent metal ions (Ca^2+^, Mg^2+^ and Mn^2+^) and chelating agent EDTA were tested and showed that rBPSL1038 is non-dependent towards metal ions. The images (**a**–**d**) were obtained from the original images edited using ImageJ software^65^ by colour inversion using lookup table, followed by enhancing contrast with saturated pixels of 0.3% before the image were cropped. The uncropped images were shown in Supplementary Fig. 7.

Due to the Cas2 similarities, we next investigated the nuclease activity of rBPSL1038 using pUC19 plasmid as substrate. The purified rBPSL1038 cleaved the plasmid in a time-based sequential degradation mode (Fig. 6a, Supplementary Fig. 7a). The supercoiled pUC19 plasmid was gradually nicked to a relaxed circular form, and eventually into a linearized form. These observations suggest that BPSL1038 can function as a dsDNA endonuclease; nonetheless, the detailed mechanism of the supercoiled DNA nicking has yet to be elucidated. We further observed that the endonuclease activity was significantly reduced when the reaction buffer contained 100 mM or more NaCl or KCl (Fig. 6b, Supplementary Fig. 7b). This suggests that the concentration of monovalent ions in the reaction buffer may act as a determining factor for nuclease activity, or the increased salt concentration may weaken nucleic acid binding, subsequently affecting nuclease activity.

The nuclease activity of rBPSL1038 for dsDNA was optimum at pH 6 (Fig. 6c, Supplementary Fig. 7c), suggesting pH-dependent activity. This differs from the previously reported Cas2 which are Xal_Cas2^26^, Bha_Cas2^10^ and Spy_Cas2^17^ where nuclease activity was either inactivated or significantly decreased at acidic pH. To further analyze if BPSL1038 is a divalent-cation-dependent nuclease, the dsDNA cleavage activity of rBPSL1038 with (Ca^2+^, Mg^2+^ and Mn^2+^) and without divalent cations as well as chelating agent (EDTA) were tested. Interestingly, rBPSL1038 cleaved dsDNA in the absence of divalent ions or in the presence of Mg^2+^, Mn^2+^, Ca^2+^ or EDTA (Fig. 6d, Supplementary Fig. 7d), suggesting that rBPSL1038 may act as a divalent-ion-independent nuclease.

**Fig. 7:**
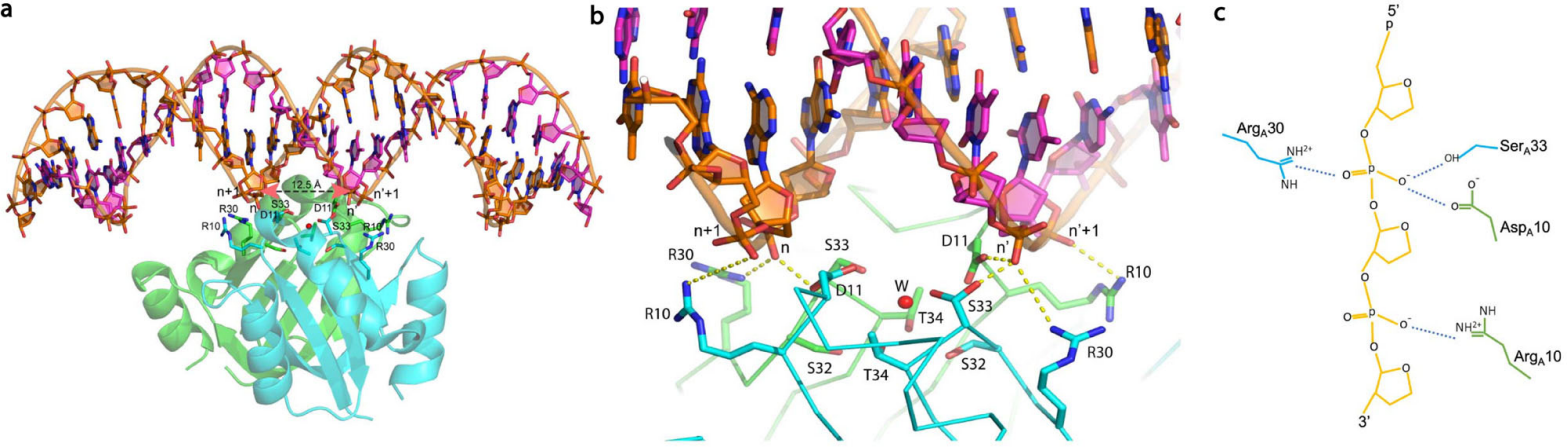
Interaction of double-stranded DNA (dsDNA) with BPSL1038 for metal ion independent cleavage analysis. **a** The manual docking of dsDNA to the proposed active site of BPSL1038. **b** Close view of two scissile phosphates (n, n’) of the minor groove that may interact with two active sites (D11_A_, S33_A_, R30_B_ and D11_B_, S33_B_, R30_A,_ respectively) with the distance of ~2.6–4.5 Å, while residue R10 is proposed to interact with the phosphate group of nucleotide n + 1, n’+1. **c** Schematic presentation of the proposed BSPL1038-DNA interaction that may initiate metal ion-independent cleavage DNAase activity.

In order to propose a basic mechanism of how BPSL1038 is able to cleave dsDNA via a metal-independent reaction, we further explored the geometry of the potential functional site involved in nucleic acid binding and cleavage. Although the structure comparisons with other Cas2 proteins have provided substantial structural similarity to support the metal ion-dependent nuclease hypothesis (Fig. 5d), further structural analysis revealed that two scissile phosphates (n, n’) of the minor groove, located 12.5 Å apart, are in position to be at the two potential active sites consisting of D11^A^, S33^A^, R30^B^ and D11^B^, S33^B^, R30^A^, respectively with distances in the range of ~2.6–4.5 Å, while residue R10 is likely to interact with phosphate group of nucleotide n + 1, n’+1 (Fig. 7).

### rBPSL1038 conformation in various salt concentrations

**Fig. 8:**
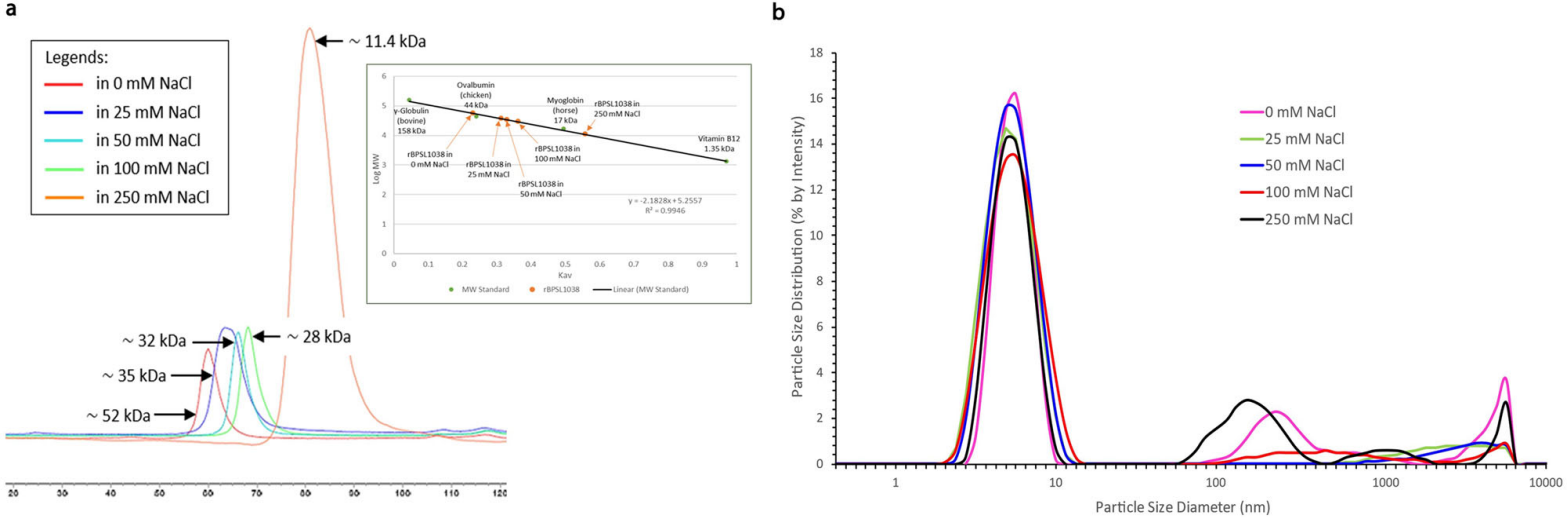
Analysis of rBPSL1038 conformation in various salt concentration. **a** Size exclusion chromatography (SEC) analysis of rBPSL1038 in buffer containing 0, 25, 50, 100 and 250 mM of NaCl using HiLoad 16/600 Superdex 75 pg column (Cytiva, USA). *X*-axis represents the retention volume of the SEC. Represented by the estimated molecular weight, the profile shows that rBPSL1038 protein undergoes conformational changes as the NaCl concentration increases. The rBPSL1038 appears as a monomeric protein (11 kDa) in 250 mM of NaCl but forms a dimer (~28 kDa) and oligomeric proteins (32, 35 and 52 kDa) with the reduction of salt concentrations. **b** Dynamic scattering analysis of rBPSL1038 size distribution in SEC buffer (25 mM Tris pH 7.5 containing 0, 25, 50, 100 and 250 mM NaCl). The diameter of all the BPSL1038 samples was measured by Zetasizer Nano Series (Malvern Panalytical, UK) with an average diameter of 5.6 nm (range of 4.8–6.5 nm), indicating that the size of the rBPSL1038 (~27–37 kD) remains similar in various salt concentrations, suggesting that the observation of NaCl concentration-related SEC profiles could be an artefact.

The calculated molecular weight of rBPSL1038 fused with a 6x-His tag is 12.1 kDa. Size exclusion chromatography (SEC) demonstrated that the molecular weight in a solution containing 100 mM NaCl was ~28 kDa, indicating that rBPSL1038 could be a dimer. Nonetheless, increasing the NaCl concentration in the SEC elution buffer significantly increased the rBPSL1038 retention volume (Fig. 8a), proposing that rBPSL1038 may undergo conformational changes in the presence of different NaCl concentrations. rBPSL1038 appeared as a monomeric protein (~11.4 kDa) in buffer containing 250 mM NaCl and shifted to a dimeric (~28 kDa) and oligomeric conformations (~32, ~35 and ~52 kDa) in the presence of 100, 50, 25 and 0 mM NaCl, respectively (Fig. 8a). To investigate the NaCl concentration-related retention volume shift of the SEC profile, the SEC-purified rBPSL1038 from various salt concentrations (100, 50, 25 and 0 mM) was subjected to DLS analysis. The results show a consistent diameter with an average of 5.6 nm (range of 4.8–6.5 nm) in all tested NaCl concentrations (Fig. 8b), indicating that the size of the rBPSL1038 (~27–37 kDa) remains similar in various salt concentrations, suggesting that the observation of NaCl concentration-related SEC profiles could be an artefact.

### BPSL1038 is non-toxic to nematodes

The genes in the CRISPR-Cas systems were found to encode numerous nucleases that are homologue to prokaryotic toxins^27^. One of these predicted toxins is the Cas2 protein, which may play a role in programmed cell death in the anti-phage defence system or when bacteria respond to environmental stresses^13,27^. The potential toxic effect of BPSL1038 on its host during infection was evaluated using a transgenic *C. elegans ugt-29::gfp* detoxification biosensor^28^ which is based on activation of the UDP-glucoronosyl transferase (UGT) detoxification mechanism in the presence of toxins and xenobiotics. The transgenic worms were fed with *E. coli* bearing the rBPSL1038 construct under IPTG-induced conditions up to 48 h or treated with crude rBPSL1038 protein for up to 96 h and fluorescence was monitored.

**Fig. 9:**
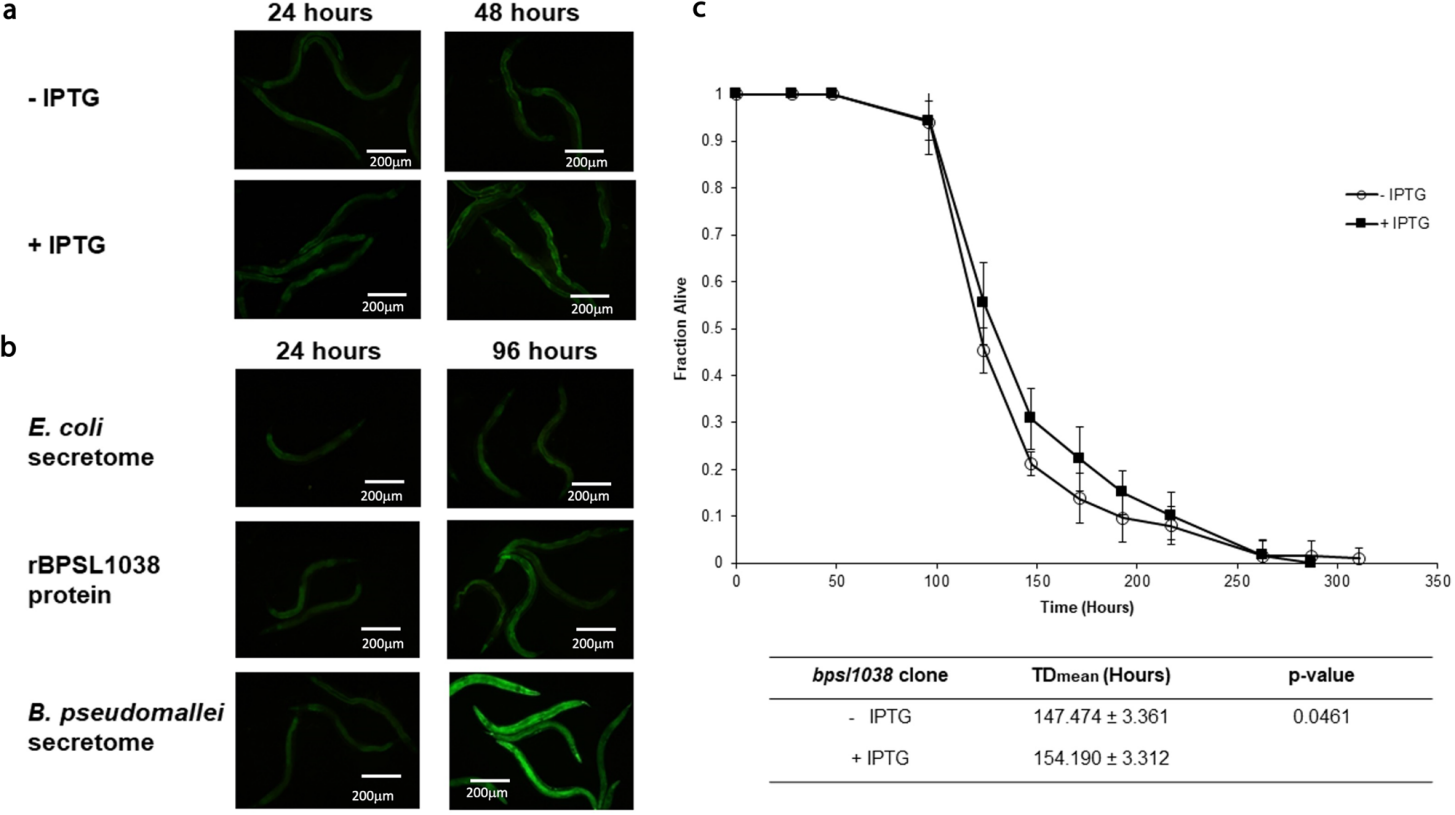
BPSL1038 does not exhibit toxin-like properties. **a** *ugt-29* is expressed only mildly in worms fed with *E. coli* bearing the rBPSL1038 construct ± IPTG over 48 h. **b** Representative fluorescence micrographs (×100 magnification) of GFP expression to visualize transcriptional activity of *ugt-29* in worms exposed to *E. coli* (negative control), recombinant protein (rBPSL1038) and *B. pseudomallei* secretome (positive control) at 24 h and 96 h. Faint expression of GFP was noted for *C. elegans ugt-29::gfp* exposed to *E. coli* and rBSL1038 at both time points while significant fluorescence was observed in worms exposed to *B. pseudomallei* secretome. Fluorescence micrographs of the same 100×magnification were acquired at the same exposure time and gain factor. **c** Nematode killing curve of worms infected by *E. coli* bearing the rBPSL1038 construct ± IPTG. The calculated mean-time-to-death (TD_mean_) is not significantly different between both treatments. Error bars represent mean value ± SD.

Worms that were fed with *E. coli* carrying the recombinant clone in the absence or presence of IPTG only showed mild fluorescence even after 48 h (Fig. 9a). Meanwhile, fluorescence was observed for worms treated with 1 mg rBPSL1038 protein although the intensity was not significantly higher when compared to control worms exposed to *E. coli* secreted proteins. Sporadic areas of fluorescence at 96 h (Fig. 9b) suggested a toxin-like property that mildly activated the detoxification system. However, the fluorescence was not as intense as that seen for worms treated with the wild-type *B. pseudomallei* secretome which is known to contain toxic molecules such as bactobolin^28^. We then performed a nematode survival assay where wild type *C. elegans* were fed with *E. coli* bearing the rBPSL1038 construct grown on NGM ± IPTG and the result showed no significant difference in mean-time-to-death (*p* > 0.0001) between worms fed on induced and uninduced recombinant clone (Fig. 9c, Supplementary Data 1). Collectively, the data ruled out the possibility that BPSL1038 is a toxin.

### Limited Cytotoxicity of rBPSL1038 against MCF7 human breast carcinoma cell

To confirm the above findings that BPSL1038 is not toxic, the MTT assay (*n* = 9) was performed on human breast cancer MCF7 cells treated with rBPSL1038. The assay demonstrated that BPSL1038 displayed limited cytotoxicity towards the MCF7 cells. The IC_20_ (20% inhibition) of MCF7 cell growth following 72 h of BPSL1038 treatment was 305 ± 10.80 µg/mL. Only 30% of cell growth was inhibited at concentrations up to 800 µg/mL (Supplementary Fig. 8 and Supplementary Table 1). Taken together, the nematode and human cell line models provide evidence that BPSL1038 is non-toxic.

## Discussion

BPSL1038 is a protein with 88 amino acids encoded by a gene annotated as hypothetical in *B. pseudomallei*. While homologs of this protein were found across six genera, no homologs were detectable in Archaea and Eukaryotic species. Orthologs of the *bpsl1038* gene are present in both pathogenic and non-pathogenic bacterial species^4^. To date, all the available homologous sequences have been annotated as either hypothetical or uncharacterized proteins.

In the absence of a characterized homolog for BPSL1038 in the Protein Data Bank (PDB), the structure of BPSL1038 was determined via experimental phasing using the SAD method with the Se-Met substituted protein crystals diffracting to 1.88 Å resolution. The crystal structure revealed that the asymmetric unit comprises of two molecules of BPSL1038 that form a homodimeric structure. The structure was further refined and modelled to 1.55 Å resolution using a good quality native protein dataset.

The molecular structure for BPSL1038 was shown to contain domains with a ferredoxin-like fold similar to several CRISPR-associated Cas2 proteins such as those from *P. furiosus* DSM 3638 (PDB ID: 4TNO and 2I0X), *T. thermophilus* (PDB ID: 1ZPW) and *T. onnurineus* (PDB ID: 5G4D). The observed fold similarity could be seen in a sequence alignment only when the fold context was provided (Fig. 2c) and was otherwise not detectable by a database search using pairwise local similarity search tools such as BLAST. This lends credence to the possibility that BPSL1038 is a member of a yet uncharacterized protein family/subfamily within the superfamily of Cas2 proteins. The discovery that BPSL1038 had structural similarities to Cas2 homologs (Supplementary Fig. 4) that were not detectable at the sequence level led to further routes of investigations to characterize the function of BPSL1038.

Substantial divergence was observed at α1, loop β1-α1, loop α2-β4, β4 and the C-terminal regions of BPSL1038 compared to Cas2 and Hpy_VapD proteins. The α1 of BPSL1038 is sandwiched between two 3_10_-helices, an arrangement that is not seen in all examples of Cas2 and Hyp_VapD. The coordinate of 3_10_-helix α-3_10_1 of BPSL1038 was located at the region equivalent to loop β1-α1 in Cas2 proteins. A long β1-α1 loop was proposed as the DNA binding feature for the Bha_Cas2, Spy_Cas2 and Xal_Cas2 proteins^10–12^. However, BPSL1038 has a rather short turn connecting the β1 and α1 through α-3_10_1 (Fig. 3a). Furthermore, BPSL1038 also lacks the long α2-β4 loop that was suggested as a signature feature for Cas2 proteins to grip ssRNA as seen in Sso_Cas2^13^; the equivalent region in the Hpy_VapD protein consists of an additional helix. Both Sso_Cas2 and Hpy_VapD are known endoribonucleases (Fig. 3b, d). Interestingly, Tth_Cas2 was found to have both β1-α1 and α2-β4 loop regions with similar dimensions as BPSL1038 (Fig. 3c). Tth_Cas2 was reported to possess dual nuclease activity towards dsDNA and ssRNA substrates but with a preference for the former^10^. This observation indicates that it may be possible that BPSL1038 functions as a nuclease like Tth_Cas2. Nonetheless, the electrostatic surface of BPSL1038 is vastly different from that of Tth_Cas2 (Supplementary Fig. 6) in keeping with the low sequence identity (11%) (Fig. 2a) which suggests that the substrate binding mechanisms for these proteins are different or that they have conserved nuclease functions but also have different components involved in very different cellular roles.

Despite the generally low sequence identity between BPSL1038 and the Cas2 homologs, multiple sequence alignment shows that the catalytically important aspartate residues in Cas2 and Hpy_VapD are indeed conserved in BPSL1038 and identified as residue D11. The coordination of residue D11 to form an aspartate pair that is similar to those in the Cas2 and Hpy_VapD proteins (Fig. 3) suggests a similar functionality. The D11 pair in BPSL1038 is 5.9 Å apart, and thus much closer compared to those identified in the Cas2 proteins that are in the range of 6.5–15 Å apart^10,12^. This makes it possible for BPSL1038 to coordinate with divalent metal ions such as Mg^2+^ that are important for Cas2 nuclease activity. Interestingly, D11 pairs together with residues S33 and T34 to constitute a negatively charged cage that coordinating a water molecule (w1) (Fig. 4). The distances of the two aspartate side chains are 3.2 and 3.6 Å from w1, indicating that with a minor conformational change, the aspartate pair may be able to coordinate with a divalent ion like Mg^2+^. Hence, it is likely that both aspartate residues may coordinate with a metal cation (Mg^2+^) to activate a nucleophilic water molecule, similar to the proposed mechanism of Sso_Cas2^13^. The activated hydroxyl group of the water molecule will then initiate a nucleophilic attack on the scissile phosphate. The negatively charged penta-coordinated transition state is possibly stabilized by the metal ion or the side chain of R30. The close proximity of the side chain hydroxyl group of S33 to the aspartate pair at 2.5 to 4.1 Å, respectively, makes the interaction with the Mg^2+^ ion possible (Fig. 5d) to likely forms an octahedral coordination. Nonetheless, the nuclease assay results indicate that rBPSL1038 is likely using a divalent-ion independent cleavage mechanism because the use of EDTA to chelate free metal ions was found to not have an effect on dsDNA cleavage activity (Fig. 6d). It is also possible that the hydroxyl group on the S33 side chain can initiate the nucleophilic attack to the phosphodiester bond, while the residues R30 can stabilize the penta-covalent intermediate (Fig. 7) similar to the metal ion independent Ser recombinases^29–31^ previously reported.

This suggests that BPSL1038 is unique as most Cas2 proteins have been classified as metallonucleases^11–13,15,32^. The divalent-ion-independent catalytic mechanism towards cleavage of RNA was previous reported for VapD^16^ and Lin_Cas2^15^, however, divalent ion-independent DNAse activity has not been described for Cas2 proteins. While divalent metal-independent RNA catalysis is possible as the 2’-hydroxyl group of ribose can serve as an intrinsic nucleophile^15^, the divalent ion-independent DNase activity requires further investigation.

Further structural analysis revealed that the D11 equivalent residue in Efa_Cas1-2/prespacer complex (PDB ID: 5XVN)^20^ together with S43^Efa_Cas2^ (T34 in BPSL1038) residues that interact with Mg^2+^ are in contact with the prespacer DNA backbone (Fig. 4b). These observations suggest that the D11 and SST^33–35^ sequence may serve as a signature motif unique to members belonging to this BPSL1038 family. This is further supported by the conservation of this motif throughout the 85 species with homologs from seven genera identified from the NCBI protein databank and Burkholderia genome databases (https://www.burkholderia.com/orthologs/list?id=371057) (Supplementary Fig. 1).

Structural superposition of BPSL1038 with the *E. coli* Cas1-Cas2-protospacer DNA^25^ and Efa_Cas1-2/prespacer complexes^20^ further revealed that dsDNA may interact with the potential D^11^ (X20)SST active site of BPSL1038 (Figs. 4b, 5). Moreover, the arginine clamp residues (R16 and R78) of Eco_Cas2 in the *E. coli* Cas1-Cas2 complex that grasp the protospacer DNA was shown to share a similar coordination with R10 and R30 of BPSL1038; suggesting that BPSL1038 may interact with the substrate DNA backbone in a similar manner (Fig. 5). Hence, we have modelled the dsDNA (matrix shown in Supplementary Table 2) onto the BPSL1038 structure using Eco_Cas2 as a reference (Fig. 5b). By changing the side chain rotamer of R10 and R30 with the guanidinium groups pointing towards the modelled DNA phosphate backbone, the arginine pairs may clamp the phosphate group of nucleotides (n3’ and n9), separated by a half turn of DNA helix across the minor groove. As a result, we hypothesize that the dsDNA is exposed to the putative active site of BPSL1038 without obvious steric clashes and the proposed active site with electronegative environment will catalyze cleavage at the nucleotides position (n4 and n8’), one position before the arginine interacting site that shows closest proximity to the D11 aspartate pair (Fig. 5b, c).

Mutation of the D11 equivalent aspartate residue in Sso_Cas2, Hpy_VapD and Lin_Cas2 have been reported as important for catalytic activity^13,15,16^. Previously, mutated residues Q33, Y34 and S35 of Sso_Cas2 with coordinates equivalent to the SST motif were also shown to slightly reduce RNAse activity. This suggests that a mutation of the highly conserved S32 residue of BPSL1038 may disrupt the structure of ST-turn that consists of residues S33 and T34 and prevent the formation of the active site (Fig. 4). It is worth noting that the SST motif that assembled the ST-turn was not found in either Sso_Cas2 or other Cas2 proteins (Fig. 2a). Taken together, BPSL1038 may have evolved to assemble a unique catalytic site compared to other known Cas2 and VapD homologs. The R10 and R30 residues that is proposed also function as a pair of arginine clamps similar to that of Eco_Cas2^36^. Although the DSST motif on its own can be associated with a known DNA binding domain (PDB ID: 3EQ5), the combined arrangement of DSST(DSST-DSST) in both protomers is a novel arrangement that has only been detected in this structure and we believe it to also be structurally conserved in members of *Burkholderiacea* in which the sequence motif is present.

The nuclease activity assays clearly demonstrate that BPSL1038 is an endonuclease capable of cleaving dsDNA (Fig. 6a). The cleavage of dsDNA is consistent with the studies reported for Spy_Cas2, Bha_Cas2 and Xal_Cas2^10–12^, Nonetheless, unlike the activities of all these known Cas2 dsDNA-targetting DNases that are divalent metal ion dependent, the activity of BPSL1038 was shown to be divalent ion independent (Fig. 6d). In Cas2 proteins, a divalent-ions-independent catalytic mechanism on RNA was previously reported for VapD^16^ and Lin_Cas2^15^, but not for divalent ion independent DNAse activity. While divalent metal-independent RNA catalysis is possible as the 2’-hydroxyl group of ribose can serve as an intrinsic nucleophile^15^, we modelled a hypothetical interaction of how the active site and associated arginine clamp residues (R10 and R30) may be able to interact with a target DNA and fit into the minor groove to allow for the catalysis reaction to occur (Fig. 7). This model is able to propose a mechanism of how the DNase activity is carried out in a divalent ion independent manner.

Unlike Hpy_VapD nuclease activity that is not influenced by monovalent ions (Na^+^ and K^+^), Bha_Cas2 and Lin_Cas2 were more active in the presence of high monovalent K^+^ and Na^+^ concentrations (50–200 mM) with K^+^ being a better metal than Na^+^^10,15,16^. An opposite monovalent cation effect was observed for rBPSL1038 where NaCl and KCl concentrations of >100 mM significantly inhibited the dsDNase activity (Fig. 6b). This points to the existence of a diverse metal ion regulatory system for dsDNase that shares a similar dimeric ferredoxin fold. Nonetheless, the exact mechanism of how the monovalent cation participates in nuclease activity is currently unknown.

In CRISPR-Cas systems, Cas2 is known to provide a scaffolding role by binding to the new spacer DNA while Cas1 acts as the nuclease during the spacer acquisition process. As a result, the biological function of nuclease activity for Cas2 proteins remains an unresolved question. Gunderson and co-workers revealed that Lpn_Cas2 nuclease activity was associated with bacterial virulence during host infection^33^, similarly, the VapD protein homolog is also known as a virulence-associated protein^16^. In *B. pseudomallei*, neither a CRISPR-Cas2 system nor a Cas1 gene homolog has been detected^34^. Hence, the revelation that BPSL1038 shares a similar structure and function to Cas2 associated proteins raises two hypotheses: (i) *B. pseudomallei* may contain other Cas genes with significantly diverged sequences that have made them undetectable by sequence similarity searches or (ii) BPSL1038 may be a virulence associated protein or toxin similar to Lin_Cas2 and VapD protein. Nonetheless, the first hypothesis may not be fully applicable as no CRISPR elements have been found in *B. pseudomallei*^34^. To address the possibility that BPSL1038 may exert a toxic effect on its host, we assessed the potency of the rBPSL1038 protein on *C. elegans* and the ability of this protein to induce the nematode detoxification system. The data indicates that BPSL1038 is most likely not a virulence-associated protein. In addition, rBPSL1038 also showed limited cytotoxicity against the human breast cancer MCF7 cell line, suggesting that BPSL1038 may not be a cytotoxic protein. Nonetheless, in the CRISPR-Cas original study, Cas2 was hypothesized to be derived from the VapD Toxin-antitoxin (TA) system^27,35^.

In conclusion, structural similarity of BPSL1038 points towards it sharing a common ancestor with Cas2 and VapD proteins. Our results show that despite structural similarity to Cas2 and VapD, only the putative arginine clamps stand out in a generally negatively charged surface that are a stark contrast to the surface of Cas2 and VapD. Furthermore, although there is substructural similarity of the DSST motif to a Ski-like DNA binding domain, the extended functional site DSST-DSST arrangement in the dimeric form is unique. The evidence thus far suggests that BPSL1038 represents a sub-family of nucleases with metal independent capacity and a yet to be determined role in the physiology of the bacterial species that produce it.

## Methods

### Protein expression and purification

The recombinant BPSL1038-selenomethionine substituted protein (smBPSL1038) was expressed, purified, and crystallized as previously reported^7^ except that the *E. coli* strain (B834) was cultured in minimal media. Briefly, bacterial cells from 20 mL overnight cultures grown in Luria Bertani (LB) media were harvested at 1664g, 4 °C for 15 mins. Excess LB was removed by resuspending the pellet in phosphate-buffered saline (PBS) buffer followed by centrifugation at 1664g, 4 °C for 15 mins. The cell pellet was then resuspended with minimal media containing selenomethionine before inoculation into 1 L selenomethionine media and grown at 37 °C until OD_600_ = 0.5–0.8. The culture was then moved to 16 °C and induced with 1 mM isopropyl-β-D-1-thiogalactopyranoside (IPTG) overnight. For functional analysis, N-terminus 6X-His tagged recombinant native BPSL1038 protein (rBPSL1038) was purified via a three-step purification method using (1st) Ni-NTA affinity, (2nd) anion exchange and (3rd) size exclusion chromatography. In brief, two grams of overexpressed cell pellet was resuspended with 20 ml of Buffer A (25 mM Tris-HCl pH 7.5, 1 M NaCl, 20 mM imidazole, 2 mM β-mercaptoethanol) and lysed by sonication^7^. The lysate was centrifuged, and the supernatant was loaded onto a Ni-NTA affinity column (5 ml Histrap™ HP, Cytiva) pre-equilibrated with Buffer A. Unbound proteins were then washed off with 50 ml Buffer A, followed by 50 ml Buffer B (25 mM Tris-HCl pH 7.5, 100 mM NaCl, 20 mM imidazole, 2 mM β-mercaptoethanol). The bound proteins were eluted with a linear gradient of 100 ml Buffer C (25 mM Tris-HCl pH 7.5, 100 mM NaCl, 500 mM imidazole, 2 mM β-mercaptoethanol). The eluate fractions containing rBPSL1038 were pooled, diluted with Buffer B to a final concentration of ~100 mM imidazole and supplemented with 1 mM EDTA. The diluted fractions were further purified with an anion exchange column (1 ml HiTrap® Q XL, Cytiva) pre-equilibrated in Buffer D (25 mM Tris-HCl pH 7.5, 100 mM NaCl, 100 mM imidazole, 2 mM β-mercaptoethanol, 1 mM EDTA) and eluted with a linear gradient of 20 ml Buffer E (25 mM Tris-HCl pH 7.5, 1.1 M NaCl, 100 mM imidazole, 2 mM β- mercaptoethanol, 1 mM EDTA). Subsequently, 1.5 ml of the eluate containing rBPSL1038 was further purified with a size exclusion column (HiPrep™ 16/60 Sephacryl® S-200 HR, Cytiva) pre-equlibrated with Buffer F (25 mM Tris-HCl pH 8, 25 mM NaCl) (Supplementary Fig. 9). Eluates containing rBPSL1038 were collected for functional analysis. In addition, purified rBPSL1038 protein was subjected to Mass Spectrometry analysis using the Q Exactive Plus Hybrid Quadrupole-Orbitrap mass spectrometer system (Thermo Scientific, MA, USA) for purity verification (Supplementary Fig. 9).

### Analysis of N-terminus His-tag cleavage rBPSL1038

The rBPSL1038 (0.44 mg) was treated with 4.4U of thrombin (10 unit/mg of protein*)* and incubated at 4 °C for 20 h. The sample was loaded onto the HisTrap HP 5 ml column (Cytiva, USA). Fractions of flowthrough that contained the N-terminus His-tag cleaved rBPSL1038 (rBPSL1038-post-cleavage) were collected. The rBPSL1038-post-cleavage sample was then subjected to size exclusion chromatography (SEC) analysis using the HiLoad 16/600 Superdex 75 pg column (Cytiva, USA) pre-equilibrated in 25 mM Tris–HCl (pH 7.5) and 100 mM NaCl. The SEC retention volume of the rBPSL1038-post-cleavage and the rBPSL1038 that contained the His-tag fusion were compared.

### Analysis of rBPSL1038 molecular size

SEC using a HiLoad 16/600 Superdex 75 pg column (Cytiva, USA)^7^ was repeated by adjusting the NaCl concentrations range from 0 mM to 250 mM. The retention volume profile from each SEC was compared, and relative molecular weights were calculated. The SEC-purified rBPSL1038 was subjected to Dynamic Light Scattering (DLS) analysis using the Zetasizer Nano Series (Malvern Panalytical, UK) to analyze the diameter of the BPSL1038 samples.

### Protein crystallization

SeMet-BPSL1038 (smBPSL1038) protein crystals were obtained using the reservoir solution consisting of 0.1 M sodium acetate (pH 4.6), 2 M sodium formate with 10 mg ml^−1^ protein in buffer containing 25 mM Tris-HCl (pH 7.5), 100 mM NaCl and 20 mM β-mercaptoethanol. Conditions were further optimized using the hanging-drop vapour-diffusion method in 24-well trays with drops made up of equal volumes of protein solution and precipitant (1:1 μl) and equilibrated against 1 ml reservoir solution at 293 K as previously reported^7^. The capacity of BPSL1038’s active site to bind divalent ions was investigated by soaking the BPSL1038 native crystal in cryoprotectant solution^7^ that contained an additional 25 mM MnCl_2_ for 3 h.

### Crystal structure determination and refinement

Diffraction data was collected for smBPSL1038 using the single anomalous dispersion (SAD) method using beamline I03 at the Diamond Light Source, UK at a wavelength of 0.97972 Å with oscillation of 0.20° and total oscillation of 180°. Diffraction data were processed in XDS^37^. The space group was further determined using Pointless^38^ and data were merged using Aimless^39^. The selenium sites were identified using HySS in the Phenix package^14,40^ which identified seven Se atom sites. The substructures were fed into the Phaser SAD pipeline^41,42^ for experimental phasing followed by Parrot for density modification^43^ and Buccaneer for automated model building^44^. An initial map with protein/solvent boundaries and secondary structure features resolved was obtained. The initial model was refined using REFMAC^45,46^ and subjected to automated model building using ARP/wARP^47^. ARP/wARP models allowed the tracing of 97 residues in one chain and 81 residues in the other chain, indicating the presence of two molecules in the asymmetric unit. Model rebuilding was carried out with COOT^48^ and refinement with REFMAC^45,46^. The refined smBPSL1038 model was used as a search model for molecular replacement using MOLREP^36^ to determine the native structure of rBPSL1038 from the X-ray diffraction dataset (1.55 Å resolution) collected at beamline I02 also at Diamond at a wavelength of 0.9795 Å^7^. The final models of both smBPSL1038 and rBPSL1038 contain residues from His-13 to Gly-87 in molecule A and Ala-2 to Gly-87 in molecule B with all residues falling in the favoured and allowed regions of the Ramachandran plot, as defined in PROCHECK^49^. The rBPSL1038 structure with the higher resolution was used for further analysis throughout this study. The coordinates and structure factors for both SeMet-BPSL1038 and native structure have been deposited in the Protein Data Bank (PDB) with the PDB codes 7VXT and 7VXR, respectively. For the MnCl_2_-soaked rBPSL1038 crystal, diffraction data was collected (2.05 Å resolution) using the in-house Rigaku MicroMax-007 HF X-ray diffractometer (Rigaku, Tokyo, Japan). The structure was determined using molecular replacement with the rBPSL1038 as the search model. The structure was refined to Rwork/Rfree of 0.2175/0.2575 (Supplementary Data 2–3). The data collection and refinement statistics are summarized in Table 1.

### Sequence and structure analysis of BPSL1038 hypothetical protein

To investigate the similarity between BPSL1038 and Cas2 proteins, detailed sequence, and structure analysis of BPSL1038 in comparison with CRISPR-associated Cas2 proteins and VapD protein were conducted. Multiple sequence alignment (MSA) was carried out using T-coffee (Expresso)^50^ which is able to incorporate protein structural information for the MSA^51^. The secondary structures were displayed together with MSA incorporated using ESPript 3.0^50^. Structure comparison of BPSL1038 against other protein structures in the PDB was conducted using DALI^8,9^. Protein Interfaces Surfaces and Assemblies (PISA)^52^ was used to analyze the protein structure interface and assembly. The image of the structure was generated using PyMol^53^ while electrostatic potential surface was generated using CCP4MG^54^. The BPSL1038, CRISPR-Cas2 associated and VapD proteins used in the sequence and structure analysis and comparison were labelled and listed throughout the manuscript as shown in Supplementary Table 3. Substructure similarity searching was carried out using the latest version of the ASSAM computer program accessed as a web application^55,56^.

### Nuclease activity assay

The circular plasmid pUC19 was used as a substrate for the nuclease assay. The plasmid was isolated from *E. coli* JM109 using the Primeway Plasmid DNA Extraction Kit (1^st^ Base, Malaysia). pUC19 was eluted and stored in deionized water at –20 °C. All nuclease activities were carried out using 10 µM of rBPSL1038 incubated with 10 nM of pUC19 in reaction buffer containing 50 mM MES monohydrate (pH 6), 50 mM NaCl and 2.5 mM MgCl_2_. The assays were performed at 37 °C for 5 h. The optimum reaction conditions for the DNase activity assay were determined by optimizing several parameters (pH, divalent ions, and salt concentration). The reaction was incubated over the course of 1 to 16 h. The optimal salt type and concentration for rBPSL1038 were determined using either NaCl or KCl at a series of concentrations from 5 mM to 200 mM. In the pH dependence assay, MES monohydrate was substituted with 50 mM of either sodium acetate (pH 4–5), Tris-HCl (pH 7–9) or glycine-NaOH (pH 10). Metal dependence assay was also done by replacing MgCl_2_ with either 2.5 mM of CaCl_2_, MnCl_2_ or chelating agent EDTA. The end reaction products were electrophoresed in 1% agarose gel for 60 mins at 110 V. The gels were stained with 1 µg/ml ethidium bromide for 30 min and de-stained in water for 5 min to enable visualization.

### Microscopic imaging of a UDP-glucuronosyltransferase-GFP reporter

Transgenic *Caenorhabditis elegans* expressing a UDP-glucuronosyltransferase-green fluorescent protein (ugt-29::GFP) construct^28^ was used as a biosensor to evaluate the potential toxicity of BPSL1038. *E. coli* strain BL21-Rosetta gami (DE3) carrying the pET-28b-BPSL1038 construct was inoculated into LB media and protein expression induced with 1 mM IPTG as described above. A second culture of non-induced cells was also produced. The bacteria were harvested and then seeded onto Nematode Growth Medium (NGM) plates in the presence or absence of 5 mM IPTG and 50 µg/ml kanamycin prior to the addition of *C. elegans*. The nematodes were rendered sterile by RNAi knockdown of the *cdc-25.1* gene as previously described^57^ to avoid spatial interference of GFP observation within the ugt-29::GFP worms resulting from the presence of nematode eggs. The gene *cdc-25.1* encodes a CDC25 phosphatase homolog which affects embryonic viability and is necessary for cell proliferation in the germ line. The *cdc-25.1* RNAi clone^58^ was cultured overnight in Luria- Bertani (LB) broth supplemented with 100 mg mL ampicillin at 37 °C. One hundred mL of a 25-fold –concentration liquid overnight culture was spotted onto NGM plates supplemented with 1 mM IPTG and incubated at room temperature for 24 h. Gravid worms were laid on *cdc-25.1* RNAi plates for 4 h and then transferred to similar plates for an additional 4 h of egg laying. Eggs were left to hatch and grow in the presence of *cdc-25.1* RNAi to produce sterile germ line proliferation-deficient (Glp) worms.

Approximately 40 transgenic worms were transferred to NGM plates pre-seeded with the rBPSL1038 induced or uninduced clones in the presence or absence of IPTG, respectively, and maintained at 25 °C throughout the assay for observation at 24 and 48 h. In a parallel experiment, 40 sterile transgenic worms were transferred to NGM plates infused with 1 mg crude rBPSL1038 protein for 24 and 96 h prior to GFP visualization. Worms on NGM infused with wild type *B. pseudomallei* or spread with *E. coli* OP50 bacteria served as controls. At the end of the assay, 10 transgenic worms were mounted on a 2% agarose pad placed on a glass slide for GFP examination. Worms were paralysed with 5 mM levamisole and observed under 100 × and 400 × magnifications with a Leica I3 long pass GFP filter (Leica Microsystems) on a Leica upright fluorescence microscope. All images were captured using the Leica DCF 310 FX digital colour camera and LAS version 3.8 software (Leica Microsystems).

### Caenorhabditis elegans survival assay

*C. elegans rrf-3(pk1426);glp-4(bn2)* worms were synchronized and grown on NGM at 25^o^C to adult stage. For infection assays, thirty age-matched worms were transferred to NGM supplemented with or without IPTG plates pre-seeded with *E. coli* strain BL21-Rosetta gami (DE3) carrying the pET-28b-BPSL1038 construct. Survival of worms was monitored until all the worms died (failed to respond to probing with a platinum wire picker).

### Cytotoxicity test for rBPSL1038 against MCF7 human breast carcinoma

The rBPSL1038 was tested using the MTT (3-[4,5-dimethylthiazol-2-yl]-2,5 diphenyl tetrazolium bromide) assay to observe MCF7 cell line (HTB-22™, ATCC, Virginia, United States) viability. MCF7 cells (5000 cells per well) were seeded into a 96-well plate on Day 1. After 24 hr, the cells were treated with BPSL1038 and incubated for 72 hr in a CO_2_ incubator. Then, 20 μl of 5 mg/ml MTT reagent (Sigma Aldrich, USA) was added into each well and the plate was incubated at 37˚C for 3 hr. After incubation, the MTT reagent was removed and 200 µl of Dimethyl Sulfoxide (DMSO, 5%) was added to each well. The 96-well plate was placed on an orbital shaker for 15 min. Absorbance was read at 570 nm (SpectraMax® ABS Plus, Molecular Devices, California, USA).

### Statistics and reproducibility

All the nuclease activity assay were conducted in duplicate, except the metal dependence assay (Fig. 6d) were conducted triplicate.

The *C. elegans* survival assay experiment was performed in triplicate. The analyses of *C. elegans* survival assay was performed using StatView version 5.0.1. *P*-value of < 0.0001 was considered as statistically significant.

To investigate the cytotoxicity of rBPSL1038 against MCF7 human breast carcinoma, the MTT assay was performed. Nine replicates were performed for each concentration of rBPSL1038 treatment, each with their respective control. Concentrations were as follows: 800, 400, 200, 100, 50, 25, 12.5, 6.25, 3.13, 1.56, 0.78, and 0.39 µg/ml. The cell viability (%) was calculated relative to untreated cells, and IC20 was determined using Microsoft® Excel. IC20 is presented as mean ± SD. For data visualization, graph was plotted using GraphPad Prism® (version 6.01). Cell viability is represented as mean ± SD (%) (*n* = 9, *p* < 0.05), with individual data points.

### Reporting summary

Further information on research design is available in the Nature Portfolio Reporting Summary linked to this article.

### Supplementary information

Supplementary Information

Description of Additional Supplementary Files

Supplementary Data 1

Supplementary Data 2

Supplementary Data 3

Reporting Summary

## Data Availability

Atomic coordinates and structure factors for the reported crystal structures have been deposited with the Protein Data Bank under accession numbers 7VXR and 7VXT. Source data underlying Fig. 9c are provided in Supplementary Data 1.
