# Supplementary material for: Structural and functional analyses of Burkholderia pseudomallei BPSL1038 reveal a Cas-2/VapD nuclease sub-family
Source: Commun Biol. 2023 Sep 8;6:920. doi: 10.1038/s42003-023-05265-4 (PMC10491678; doi:10.1038/s42003-023-05265-4)
Supplement: Supplementary file 1 — Supplementary Information [file 42003_2023_5265_MOESM1_ESM.pdf]

Supplementary Information

Structural and functional analyses of *Burkholderia pseudomallei* BPSL1038 reveal a Cas-2/VapD nuclease sub-family

Sofiyah Shaibullah[1‡], Nurshahirah Shuhaimi[1‡], De-Sheng Ker[1#], Nurhikmah Mohd-Sharif[1], Kok Lian Ho[2], Aik-Hong Teh[3], Jitka Waterman[4], Thean-Hock Tang[5], Rui-Rui Wong[6†], Sheila Nathan[6], Rahmah Mohamed[6], Min Jia Ng[7], Shin-Yee Fung[7], Mohd Anuar Jonet[8], Mohd Firdaus-Raih[1,9], and Chyan Leong Ng[1*]

[1]*Institute of Systems Biology, Universiti Kebangsaan Malaysia, 43600 UKM Bangi, Selangor, Malaysia*
[2]*Department of Pathology, Faculty of Medicine and Health Sciences, Universiti Putra Malaysia, 43400 UPM Serdang, Selangor, Malaysia*
[3]*Centre for Chemical Biology, Universiti Sains Malaysia, 11900 Bayan Lepas, Penang, Malaysia*
[4]*Diamond Light Source, Harwell Science & Innovation Campus, Didcot, Oxfordshire, OX11 0DE, UK*
[5]*Advanced Medical and Dental Institute, Universiti Sains Malaysia, Pulau Pinang, Malaysia*
[6]*Department of Biological Sciences and Biotechnology, Faculty of Science and Technology, Universiti Kebangsaan Malaysia, 43600 UKM Bangi, Selangor, Malaysia*
[7]*Medicinal Mushroom Research Group (MMRG), Department of Molecular Medicine, Faculty of Medicine, University of Malaya, Kuala Lumpur, Malaysia*
[8]*Malaysia Genome and Vaccine Institute, National Institutes of Biotechnology Malaysia (NIBM), Jalan Bangi, 43000 Kajang, Selangor, Malaysia*
[9]*Department of Applied Physics, Faculty of Science and Technology, Universiti Kebangsaan Malaysia, 43600 UKM Bangi, Selangor, Malaysia*

[*] To whom correspondence should be addressed. Tel: +60389214561; Fax: +60389213398

Email: clng@ukm.edu.my

[‡] These authors contributed equally to this work.
[†]Present address: *Faculty of Health and Life Sciences, Inti International University, Persiaran Perdana BBN, 71800 Nilai, Negeri Sembilan, Malaysia*
[#] Present address: *Department of Biochemistry, University of Cambridge, Cambridge CB2 1GA, UK*

This file contains:
Supplementary Figures 1 to 9
Supplementary Tables 1 to 3

Supplementary Figures:

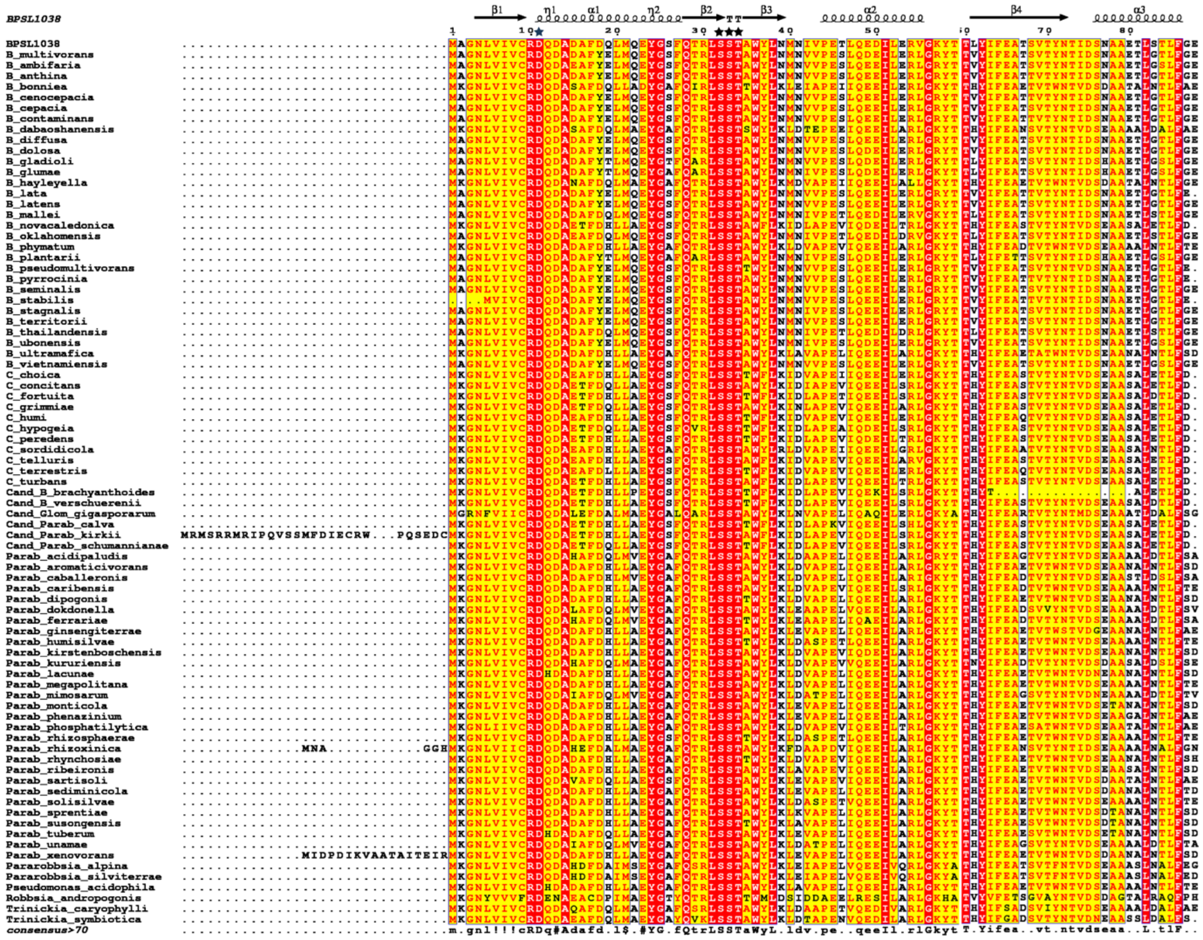

Supplementary Figure 1: Multiple sequence alignment (MSA) of 84 BPSL1038 gene homologs across seven genus (31 species of *Burkholderia* (B_), 11 species of *Caballeroni* (C_), 29 species of *Paraburkholderia* (Parab_), two species of *Pararobbsia*, three species of *Trinickia,* Pseudomonas *acidophila* and *Robbsia andropogonis* and six species *of Candidatus* (Cand_). The proposed nuclease catalytic active site (★) of BPSL1038 that contains the D11 aspartate pair and SST motif is conserved throughout all the species. The strictly conserved residues are in white characters on a red background, while similar residues are boxed yellow. The species and gene homologs of BPSL1038 used in the MSA analysis are as follows: *Burkholderia multivorans* ATCC 17616, Bmul_0891; *Burkholderia ambifaria* AMMD, BAMB_RS12360 ; *Burkholderia anthina* MSMB0848, WS84_RS33370 ; *Burkholderia bonniea*, WP_153075707.1; *Burkholderia cenocepacia* J2315, BCAL2604; *Burkholderia cepacia* UCB 717, APZ15_RS17290; *Burkholderia contaminans* FFH2055, WR30_RS04035; *Burkholderia dabaoshanensis*, WP_102646448.1; *Burkholderia diffusa* INT4-BP16, WI69_RS03505; *Burkholderia dolosa* AU0158, AK34_RS16420; *Burkholderia gladioli* ATCC 10248, BM43_RS19790; *Burkholderia glumae* BGR1, bglu_1g27730; *Burkholderia hayleyella*, WP_153099902.1; *Burkholderia lata* 383, BCEP18194_RS18815; *Burkholderia latens* RF32-BP12, WI41_RS27795; *Burkholderia mallei* 092700E, BM44_RS17955; *Burkholderia novacaledonica*, WP_106855620.1; *Burkholderia oklahomensis* C6786, BG90_RS02700; *Burkholderia phymatum* STM815, Bphy_2158; *Burkholderia plantarii* ATCC 43733, bpln_RS14270; *Burkholderia pseudomultivorans* MSMB0607,

WS80_RS09530; *Burkholderia seminalis* FL-5-5-10-S1-D0, WJ13_RS25080; *Burkholderia stabilis*, GAU00873.1; *Burkholderia stagnalis* MSMB1135, WT10_RS14690; *Burkholderia territorii* MSMB0579, WS79_RS30510; *Burkholderia thailandensis* E264 ATCC 700388, BTH_I0895; *Burkholderia ubonensis* MSMB0011, WJ31_RS01625; *Burkholderia ultramafica*, CAB3790440.1; *Burkholderia vietnamiensis* G4, Bcep1808_2488; *Caballeronia choica*, WP_087644278.1; *Caballeronia concitans*, WP_040051887.1; *Caballeronia fortuita*, WP_061135663.1; *Caballeronia grimmiae*, WP_035960696.1; *Caballeronia humi*, WP_087668243.1; *Caballeronia hypogeia*, WP_061166771.1; *Caballeronia peredens*, SAL17919.1; *Caballeronia sordidicola*, WP_060817063.1; *Caballeronia telluris*, WP_087629038.1; *Caballeronia terrestris*, WP_087655099.1; *Caballeronia turbans*, SAL51060.1; *Candidatus Burkholderia brachyanthoides*, KNH08978.1; *Candidatus Burkholderia verschuerenii*, WP_050454769.1; *Candidatus Glomeribacter gigasporarum*, WP_065379293.1; *Candidatus Paraburkholderia calva*, KMY86765.1; *Candidatus Paraburkholderia kirkii*, KND55193.1 BPUN_3165; *Candidatus Paraburkholderia schumannianae*, KND58057.1 BSCH_02360c; *Paraburkholderia acidipaludis*, WP_027795589.1; *Paraburkholderia aromaticivorans*, WP_095420447.1; *Paraburkholderia caballeronis*, WP_090545768.1; *Paraburkholderia caribensis*, WP_054929957.1; *Paraburkholderia dipogonis*, WP_134456793.1; *Paraburkholderia dokdonella*, WP_129561485.1; *Paraburkholderia ferrariae*, WP_028226141.1; *Paraburkholderia ginsengiterrae*, WP_064272291.1; *Paraburkholderia humisilvae*, CAB3773556.1 LMG29542_07310; *Paraburkholderia kirstenboschensis*, WP_128588674.1; *Paraburkholderia kururiensis*, WP_017773215.1; *Paraburkholderia lacunae*, WP_115104224.1; *Paraburkholderia megapolitana*, WP_091018721.1; *Paraburkholderia mimosarum*, WP_028210513.1; *Paraburkholderia monticola*, WP_062126742.1; *Paraburkholderia phenazinium*, WP_090686824.1; *Paraburkholderia phosphatilytica*, WP_118183806.1; *Paraburkholderia rhizosphaerae*, WP_134193879.1; *Paraburkholderia rhizoxinica* HKI 454, CBW75805.1; *Paraburkholderia rhynchosiae*, WP_102630247.1; *Paraburkholderia ribeironis*, WP_094779953.1; *Paraburkholderia sartisoli*, WP_090531588.1; *Paraburkholderia sediminicola*, WP_120303571.1; *Paraburkholderia solisilvae*, CAB3754968.1; *Paraburkholderia sprentiae*, WP_027197056.1; *Paraburkholderia susongensis*, WP_085484400.1; *Paraburkholderia tuberum*, WP_090803564.1; *Paraburkholderia unamae*, WP_112172653.1; *Paraburkholderia xenovorans* LB400, ABE29600.1 Bxe_A3385; *Pararobbsia alpina*, CAB3781688.1 LMG28138_01284; *Pararobbsia silviterrae*, WP_121083851.1; *Pseudomonas acidophila*, WP_096722440.1; *Robbsia andropogonis*, WP_024902061.1; *Trinickia caryophylli*, WP_085230237.1; *Trinickia* sp. 7GSK02, WP_136897885.1; *Trinickia symbiotica*, WP_018443060.1

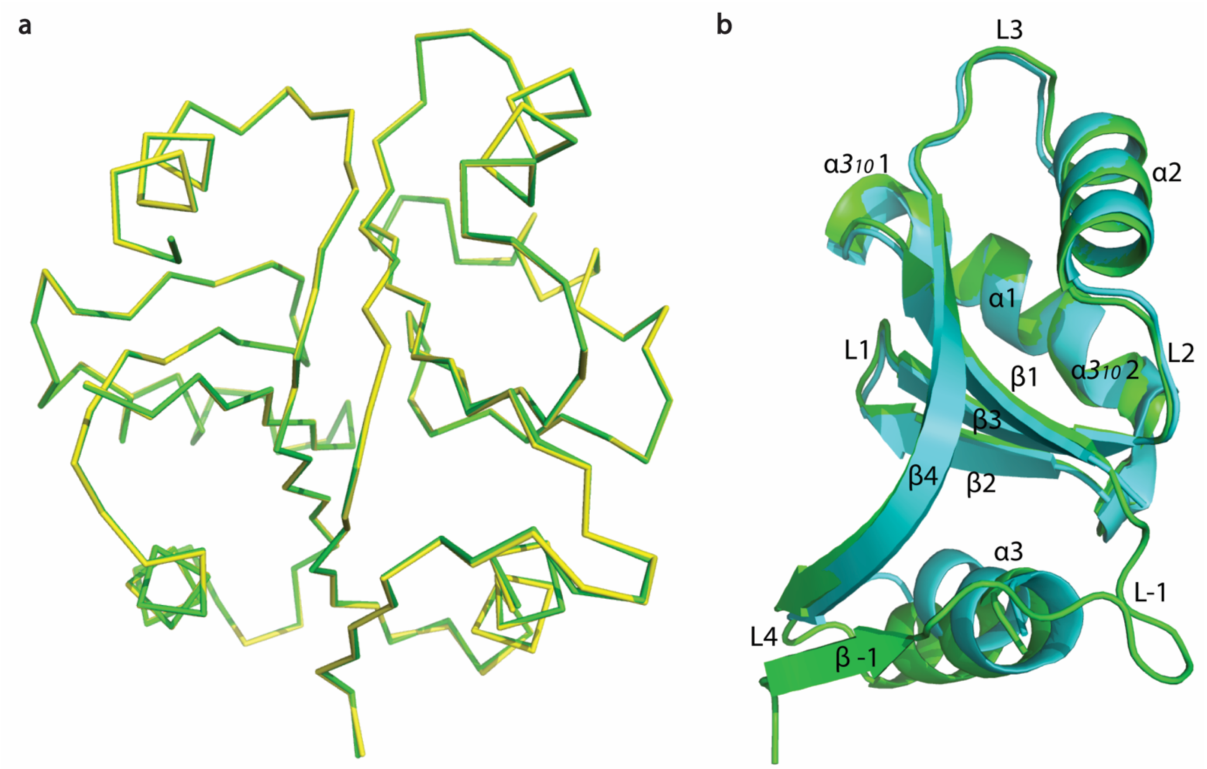

Supplementary Figure 2: Comparison of BPSL1038 crystal structures. **a** Superimposition of the homodimeric native protein BPSL1038 (rBPSL1038, green) and selenomethionine substituted BPSL0138 (smBPSL1038, yellow) shows an almost identical structure with an r.m.s.d. of 0.15 Å over 187 Cα atoms. **b** Superimposition of protomer A (green) and B (cyan) of homodimeric rBPSL1038 show a highly similar structure with an r.m.s.d. of 1.3 Å over 87 Cα atoms with the largest deviation observed at L4 loop and α3 helix. The figures were generated using Pymol[53].

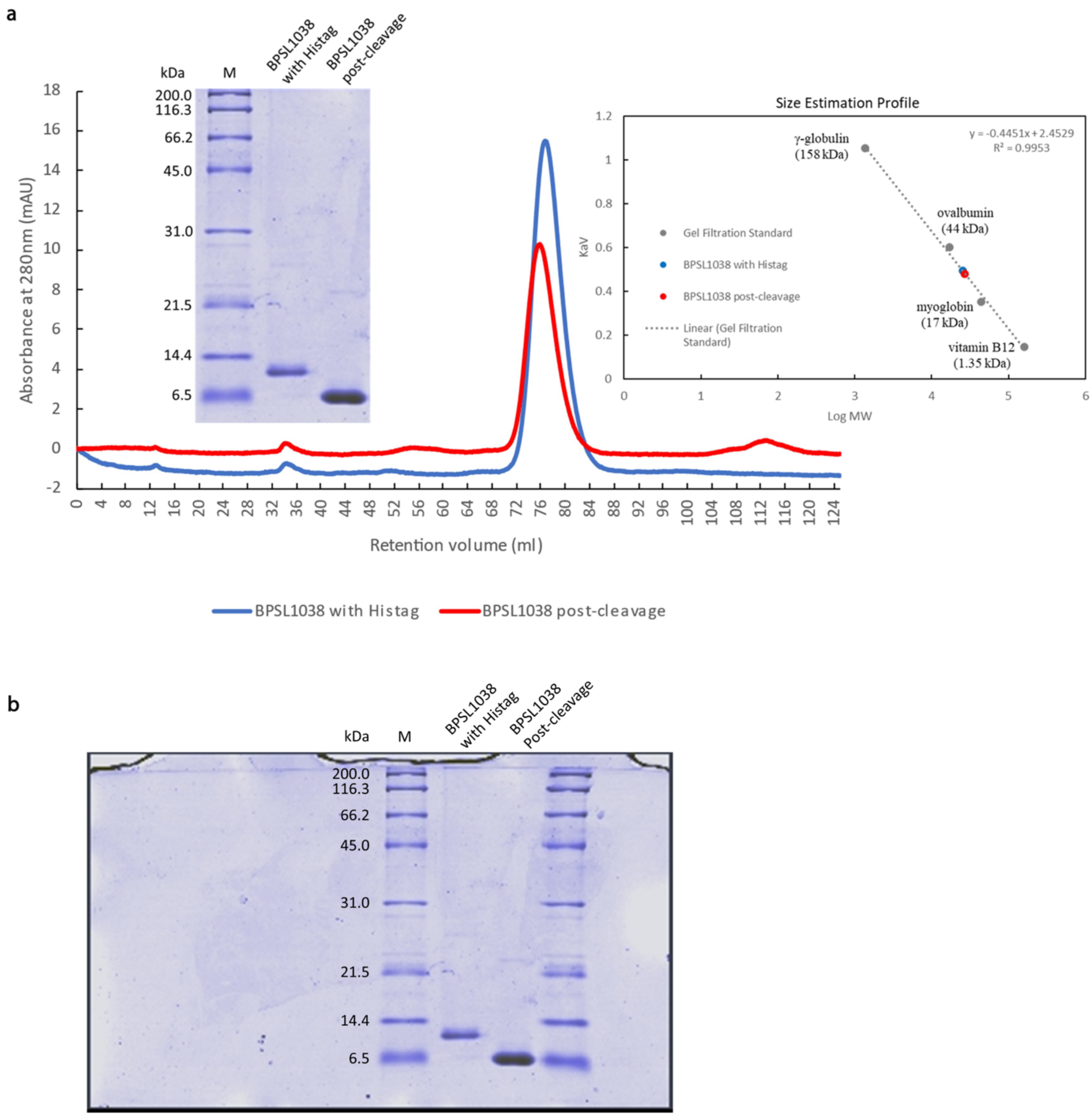

Supplementary Figure 3: The size exclusion chromatograms (SEC) analysis of the rBPSL1038 and the post-thrombin cleavage rBPSL1038 protein. **a** The SEC results indicate the retention volume of post-thrombin cleavage rBPSL1038 (without 6x-His tag fusion) and the rBPSL1038 (with 6x-His tag fusion) are similar, suggesting that no significant conformation change of the protein upon the cleavage of N-terminal fusion tag. The SDS-PAGE shows the elution fraction of the rBPSL1038 protein pre- and post-thrombin cleavage. **b** The uncropped and unedited gel image shown in **a**.

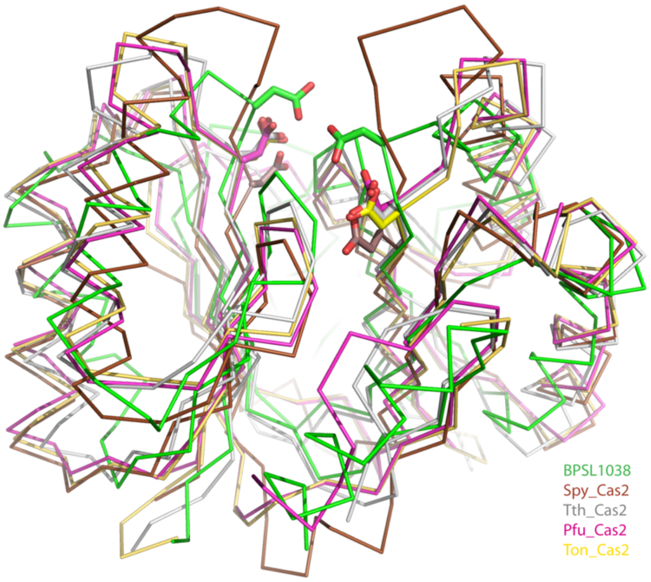

Supplementary Figure 4: Structure comparison of dimeric BPSL1038 on the DALI server identified CRISPR-associated Cas2 proteins. The SSM superimpose shows the structural similarity between BPSL1038 (green), *Streptococcus pyogenes* serotype M1 (PDB ID 4QR2, r.m.s.d 3.3 Å over 135 aligned Cα atoms); *Thermus thermophilus* (PDB ID 1ZPW, 2.5 Å r.m.s.d over 142 aligned Cα atoms); *Pyrococcus Furiosus* (PDB ID 4TNO, r.m.s.d of 2.9 Å over 144 aligned Cα atoms) and *Thermococcus Onnurineus* (PDB ID 5G4D, r.m.s.d 2.9 Å over 135 aligned Cα atoms).

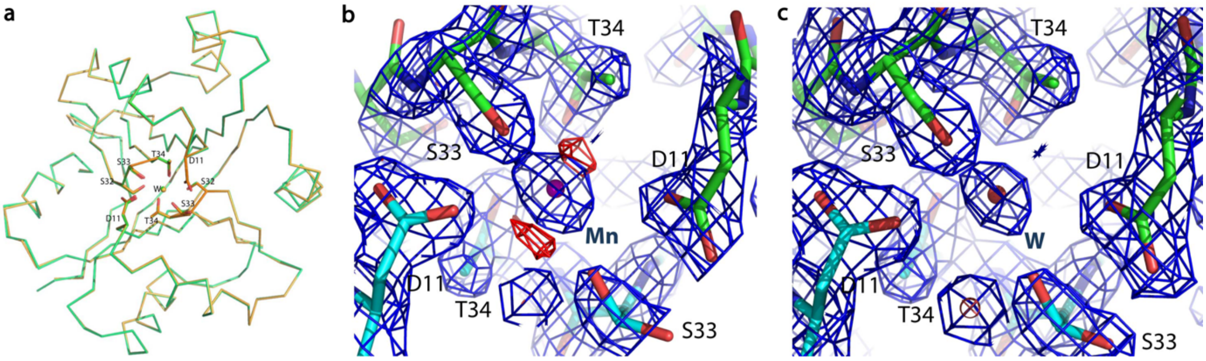

Supplementary Figure 5: Comparison of the rBPSL1038 crystal structure in the cryoprotectant condition with and without manganese chloride. **a** The SSM superimpose of rBPSL1038 (Green) to the crystal structure soaked with manganese (Orange) shows an RMSD of 0.165 Å for 186 aligned residues. **b** & **c** The 2Fo-Fc electron density map of the proposed catalytic site in the crystal structure of BPSL1038 soaked with 25mM manganese contoured at 1.0σ. The crystal structure was refined with manganese (**b**) and a water molecule (**c**) in the active site. The Fo-Fc difference map of (**b**) contoured at 3.0 σ shows negative density for the manganese ion. The results indicate that the water molecule was not substituted by cation divalent ion after three hours of soaking with 25 mM manganese chloride.

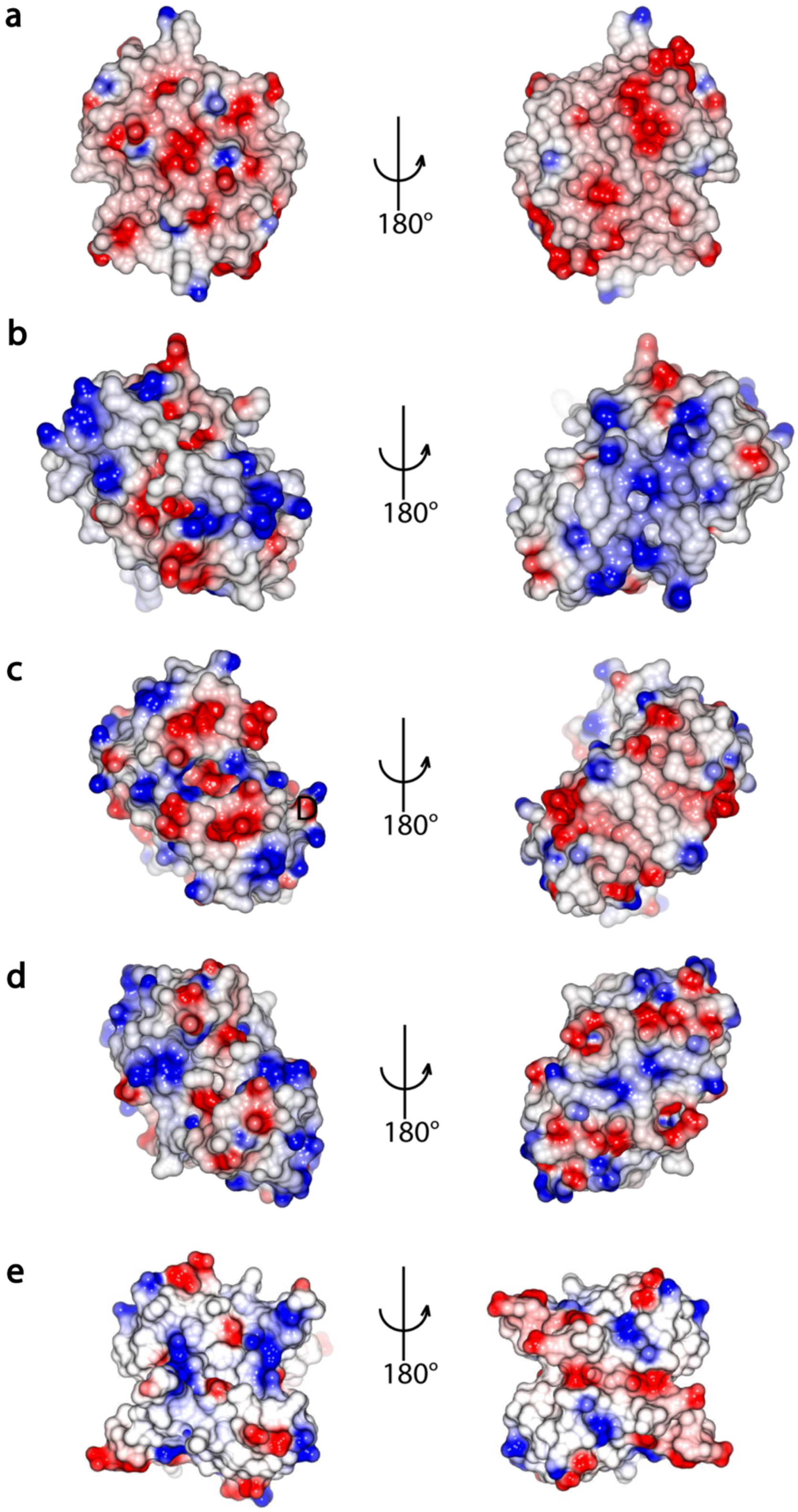

Supplementary Figure 6: Electrostatic surface of BPSL1038 (**a**), Bha_Cas2 (**b**), Sso_Cas2 (**c**), Tth_Cas2 (**d**) and Eco_Cas2 (**e**). The orientation of BPSL1038 is in accordance to Figure 1. The Bha_Cas2, Sso_Cas2, Tth_Cas2 and Eco-Cas2 are in equivalent orientation as BPSL1038. The Electrostatic potential was calculated and coloured in the range of 0.5V (Blue) to -0.5V (Red) using CCP4MG[54].

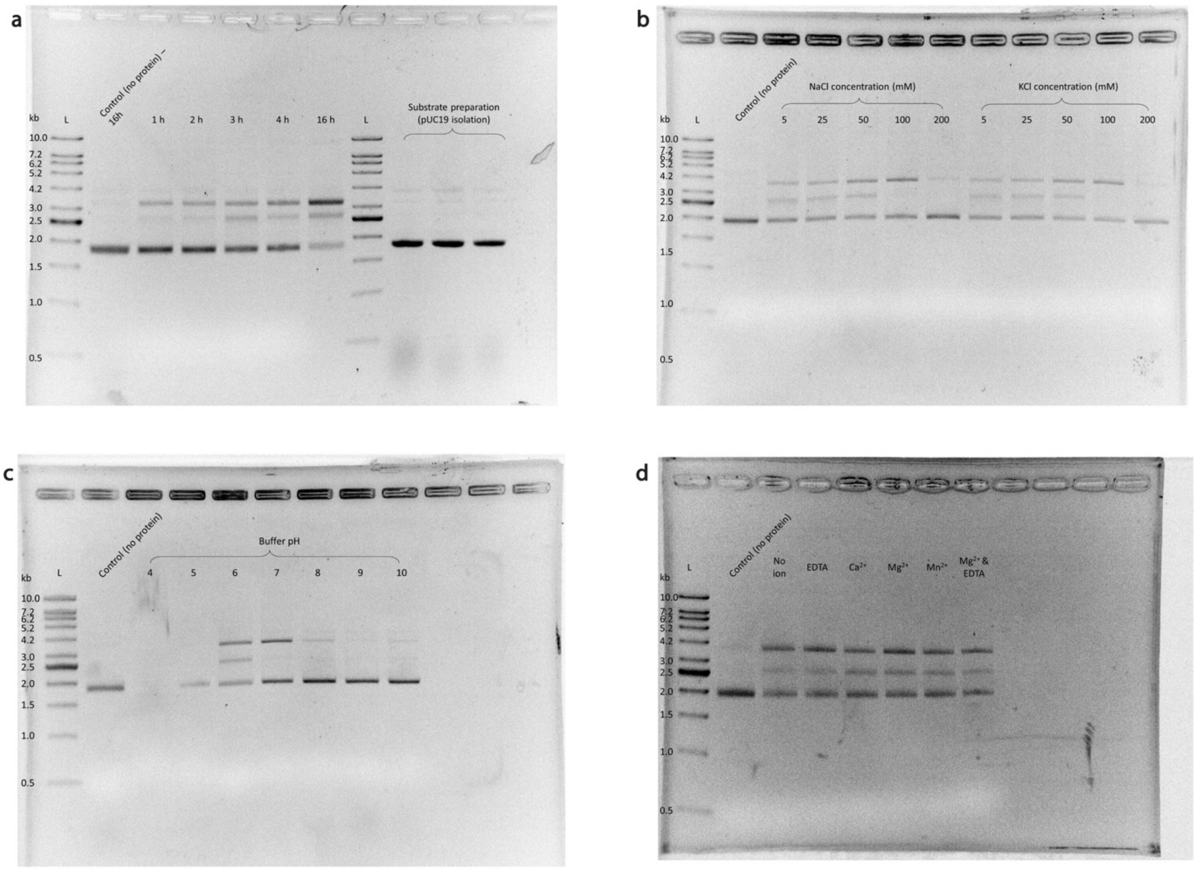

Supplementary Figure 7: The uncropped gel images for the nuclease activity assay of rBPSL1038 shown in Figure 6 (a-d).

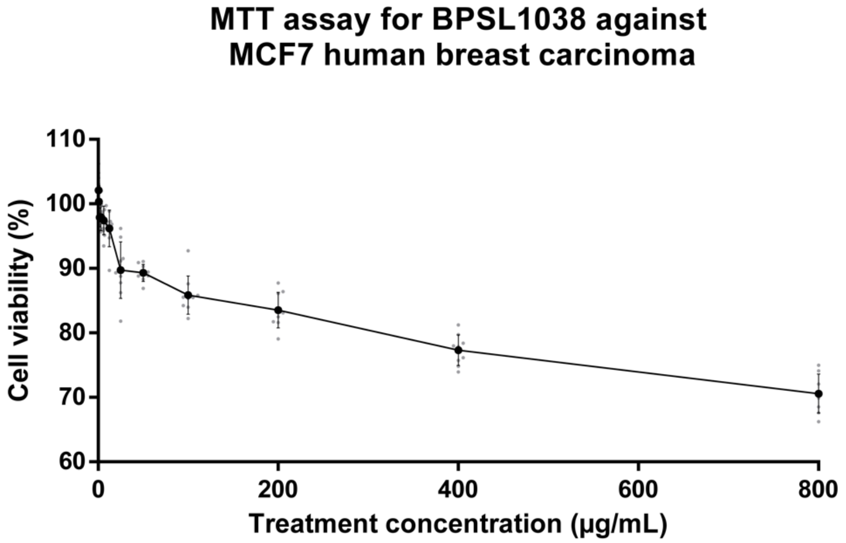

Supplementary Figure 8: Plot of cell viability (%) of breast cancer cell line (MCF7) following a 72-hour treatment with rBPSL1038, determined by MTT assay. Cell viability is represented as mean ± SD (%) ($n = 9$, $p < 0.05$).

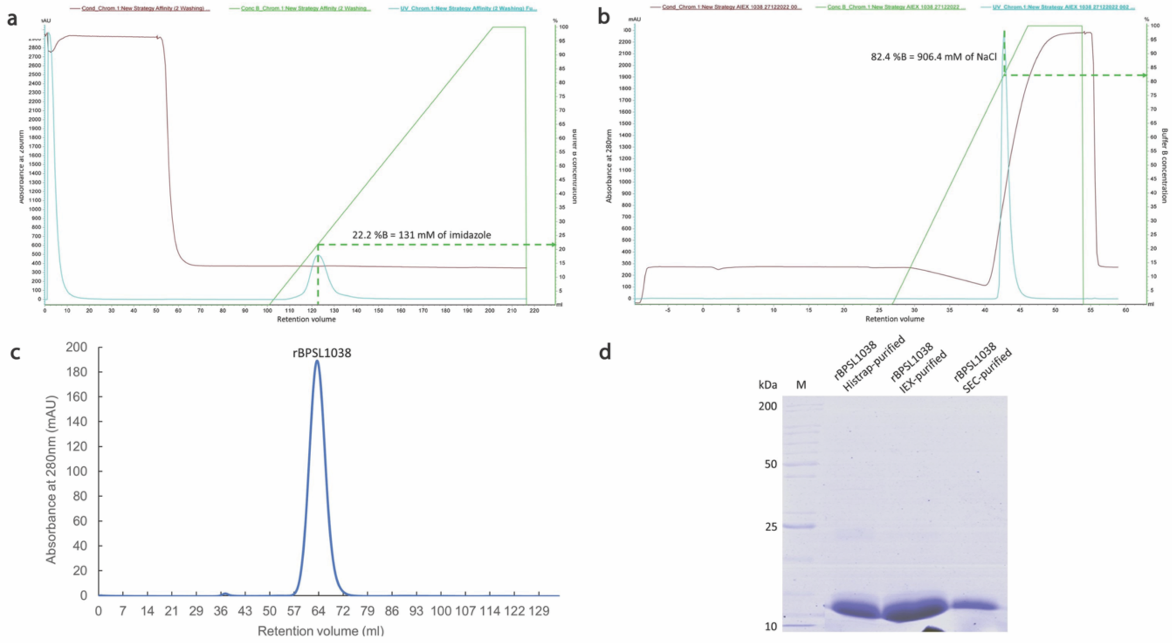

e

Description	Coverage [%]	# Peptides	# PSMs	# Unique Peptides	# AAs	MW [kDa]
protein BPSL1038	97	29	1074	29	88	9.9
Ribosome assembly factor mrt4 (Monoraphidium neglectum)	4	2	52	2	313	34
Fimbrial protein (Escherichia coli)	17	2	3	2	179	19
4-hydroxy-tetrahydrodipicolinate reductase (Alphaproteobacteria bacterium)	11	2	3	2	270	27
Ribosomal small subunit pseudouridine synthase A (Escherichia coli, strain K12)	13	2	2	2	231	25.8

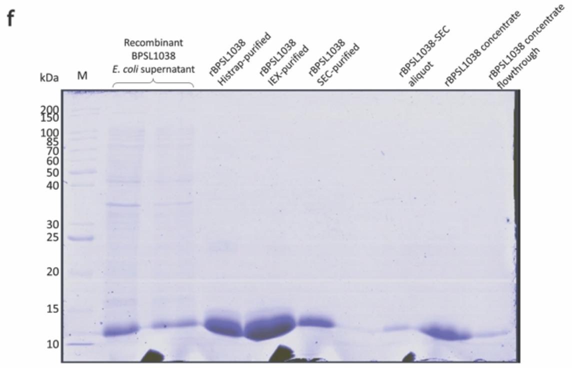

Supplementary Figure 9: Three step protein purification of rBPSL1038 using **a** Ni-NTA affinity, **b** anion exchange and **c** size exclusion chromatography. In solution, rBPSL1038 was shown to be a dimer, estimated at 28 kDa. **d** SDS-PAGE analysis of each purified step showed a distinct band at ~12 kDa, which indicates rBPSL1038. **e** Mass spectrometry analysis of rBPSL1038 post-purification searched against BPSL1038 and *Escherichia coli* sequences (unique sequence ≥ 2, molecular weight < 38 kDa), indicating that rBPSL1038 is in high purity with no other contaminant nuclease detected in the Mass Spectrophotometry analysis. **f** The unedited gel image of (**d**).

Supplementary Table 1: Cell viability (%) of MCF7 following a 72-hour treatment of rBPSL1038.

CONCENTRATION (µg/ml)	CELL VIABILITY (%)									Mean	SD
	1	2	3	4	5	6	7	8	9		
800	74.962	72.043	69.160	69.89	74.09	72.68	66.246	67.597	68.537	70.578	2.279
400	79.735	77.998	73.922	77.87	75.72	76.13	74.797	78.403	81.211	77.308	2.052
200	81.590	87.747	83.091	86.41	81.71	82.47	83.560	79.049	86.085	83.524	2.604
100	85.502	84.214	83.965	85.47	85.37	87.59	82.209	85.827	92.710	85.873	1.287
50	89.106	88.822	88.326	91.05	88.84	86.91	90.290	90.901	89.421	89.296	1.338
25	91.540	86.234	91.162	87.77	94.86	88.78	81.798	96.175	89.327	89.738	3.097
12.5	97.288	98.867	94.744	99.09	96.87	89.69	97.239	96.688	95.165	96.183	3.507
6.25	96.178	95.154	96.866	99.71	97.96	99.88	93.463	98.617	99.145	97.441	1.913
3.125	95.865	97.408	95.672	98.27	102.04	99.66	95.944	97.683	99.001	97.948	2.420
1.5625	96.094	97.275	96.745	98.91	100.05	98.19	98.773	95.956	99.073	97.896	1.463
0.78125	101.917	102.025	98.083	102.31	102.02	102.78	98.414	99.864	95.789	100.356	1.714
0.390625	102.037	103.050	99.373	104.28	104.00	101.87	106.217	100.691	97.287	102.089	1.794

Supplementary Table 2: Matric that imposed on *E. coli* Cas1-Cas2-protospacer DNA (Chain G and H) (PDB ID: 5DS5) to generate modelled dsDNA with the minor groove that is interacting with the proposed BPSL1038 'arginine clamp'.

0.98350482	0.13327226	-0.12229786	-4.19741328
-0.02005082	0.75227946	0.65853897	-0.22009183
0.17976715	-0.64522408	0.74254270	24.84206113

Supplementary Table 3: The Cas2 and VapD proteins that were found share high structure similarity to BPSL1038.

	Cas2 structures	Organism	% sequence identity	PDB Id	LAli/RMSD (Å)
1	Pfu_Cas2	*Pyrococcus furiosus*	9	2i0x	74/3.1
2	Ton_Cas2	*Thermococcus Onnurineus*	10	5g4d	72/3.0
3	Spy_Cas2	*Streptococcus Pyogenes*	6	4qr2	70/2.9
4	Dvu_Cas2	*Desulfovibrio Vulgaris*	11	3oq2	83/3.1
5	Eco_Cas2	*Escherichia Coli*	11	5dqt	81/3.1
6	Xal_Cas2	*Xanthomonas albilineans*	6	5h1p	69/2.7
7	Hpy_VapD	*Helicobacter pylori*	7	3ui3	71/3.5
