# Supplementary material for: Structural and functional analyses of Burkholderia pseudomallei BPSL1038 reveal a Cas-2/VapD nuclease sub-family
Source: Commun Biol. 2023 Sep 8;6:920. doi: 10.1038/s42003-023-05265-4 (PMC10491678; doi:10.1038/s42003-023-05265-4)
Supplement: Supplementary file 2 — Description of Additional Supplementary Files [file 42003_2023_5265_MOESM2_ESM.docx]

**Description of Additional Supplementary Files**

**File name**: Supplementary Data 1

**Description**: The source data used to plot the nematode killing curve of worms infected by *E. coli* bearing the rBPSL1038 construct ± IPTG in Figure 9c.

**File name**: Supplementary Data 2

**Description**: The coordinates for the native structure of rBPSL1038 **(**soaked with 25mM manganese), with a water molecule modelled and refined in the D^11^(X20)SST active site.

**File name**: Supplementary Data 3

**Description**: The structure factors for the native structure of rBPSL1038 (soaked with 25mM manganese), with a water molecule modelled and refined in the D^11^(X20)SST active site.
