# Supplementary material for: Structural and functional analyses of Burkholderia pseudomallei BPSL1038 reveal a Cas-2/VapD nuclease sub-family
Source: Commun Biol. 2023 Sep 8;6:920. doi: 10.1038/s42003-023-05265-4 (PMC10491678; doi:10.1038/s42003-023-05265-4)
Supplement: Supplementary file 6 — Reporting Summary [file 42003_2023_5265_MOESM6_ESM.pdf]

Corresponding author(s): Chyan Leong Ng

Last updated by author(s): 11 August 2023

Reporting Summary

Nature Portfolio wishes to improve the reproducibility of the work that we publish. This form provides structure for consistency and transparency in reporting. For further information on Nature Portfolio policies, see our Editorial Policies and the Editorial Policy Checklist.

Statistics

For all statistical analyses, confirm that the following items are present in the figure legend, table legend, main text, or Methods section.

n/a	Confirmed	
☐	☒	The exact sample size (*n*) for each experimental group/condition, given as a discrete number and unit of measurement
☐	☒	A statement on whether measurements were taken from distinct samples or whether the same sample was measured repeatedly
☐	☒	The statistical test(s) used AND whether they are one- or two-sided<br>*Only common tests should be described solely by name; describe more complex techniques in the Methods section.*
☒	☐	A description of all covariates tested
☒	☐	A description of any assumptions or corrections, such as tests of normality and adjustment for multiple comparisons
☐	☒	A full description of the statistical parameters including central tendency (e.g. means) or other basic estimates (e.g. regression coefficient) AND variation (e.g. standard deviation) or associated estimates of uncertainty (e.g. confidence intervals)
☒	☐	For null hypothesis testing, the test statistic (e.g. *F*, *t*, *r*) with confidence intervals, effect sizes, degrees of freedom and *P* value noted<br>*Give P values as exact values whenever suitable.*
☒	☐	For Bayesian analysis, information on the choice of priors and Markov chain Monte Carlo settings
☒	☐	For hierarchical and complex designs, identification of the appropriate level for tests and full reporting of outcomes
☒	☐	Estimates of effect sizes (e.g. Cohen's *d*, Pearson's *r*), indicating how they were calculated

Our web collection on statistics for biologists contains articles on many of the points above.

Software and code

Policy information about availability of computer code

Data collection	X-ray diffraction data (I03 at the Diamond Light Source, UK, Rigaku MicroMax-007 HF, MGVI, Malaysia), Mass spectrometry (Exactive Plus Hybrid Quadrupole-Orbitrap mass spectrometer system), Fluorescence microscope image (Leica 216 DCF 310 FX digital colour camera), Dynamic Light Scattering (DLS) (Zetasizer Nano Series), MTT assay absorbance (SpectraMax® ABS Plus)
Data analysis	Diffraction data and structure determination and modelling (XDS, Pointless, Aimless. Phenix, Parrot, Buccaneer, REFMAC, COOT, ARP/wARP, MOLREP,PROCHECK), Sequence analysis (Expresso, ESPript 3.0), Structure analysis (PISA, DALI, ASSAM), figure image generating (PyMol, CCP4MG), Fluorescence microscope imaging (LAS version 3.8), StatView version 5.0.1,

For manuscripts utilizing custom algorithms or software that are central to the research but not yet described in published literature, software must be made available to editors and reviewers. We strongly encourage code deposition in a community repository (e.g. GitHub). See the Nature Portfolio guidelines for submitting code & software for further information.

Data

Policy information about availability of data

All manuscripts must include a data availability statement. This statement should provide the following information, where applicable:

- Accession codes, unique identifiers, or web links for publicly available datasets
- A description of any restrictions on data availability
- For clinical datasets or third party data, please ensure that the statement adheres to our policy

Provide your data availability statement here.

Human research participants

Policy information about studies involving human research participants and Sex and Gender in Research.

Reporting on sex and gender	This study does not involve human research.
Population characteristics	This study does not involve human research.
Recruitment	This study does not involve human research.
Ethics oversight	This study does not involve human research.

Note that full information on the approval of the study protocol must also be provided in the manuscript.

Field-specific reporting

Please select the one below that is the best fit for your research. If you are not sure, read the appropriate sections before making your selection.

☒ Life sciences ☐ Behavioural & social sciences ☐ Ecological, evolutionary & environmental sciences

For a reference copy of the document with all sections, see nature.com/documents/nr-reporting-summary-flat.pdf

Life sciences study design

All studies must disclose on these points even when the disclosure is negative.

Sample size	Not relevant.
Data exclusions	No data exclusion.
Replication	The experiments (Caenorhabditis elegans survival assay and Cytotoxicity test using MCF7 human breast carcinoma) were conducted in triplicate manner. Duplicate nuclease activity assay were conducted, nonetheless, for the assay contain no ion/ions together with EDTA were conducted triplicate or more.
Randomization	Not relevant
Blinding	Not relevant

Reporting for specific materials, systems and methods

We require information from authors about some types of materials, experimental systems and methods used in many studies. Here, indicate whether each material, system or method listed is relevant to your study. If you are not sure if a list item applies to your research, read the appropriate section before selecting a response.

Materials & experimental systems

n/a	Involved in the study	
☒	☐	Antibodies
☐	☒	Eukaryotic cell lines
☒	☐	Palaeontology and archaeology
☐	☒	Animals and other organisms
☒	☐	Clinical data
☒	☐	Dual use research of concern

Methods

n/a	Involved in the study	
☒	☐	ChIP-seq
☒	☐	Flow cytometry
☒	☐	MRI-based neuroimaging

Eukaryotic cell lines

Policy information about cell lines and Sex and Gender in Research

Cell line source(s)	American Type Culture Collection (ATCC; Manassas, VA, USA)
Authentication	Cell lines from ATCC are authenticated prior to purchase by comparing the STR profile of sample cell lines with the ATCC Human Cell STR Database
Mycoplasma contamination	Not tested.
Commonly misidentified lines (See ICLAC register)	Not applicable.

Animals and other research organisms

Policy information about studies involving animals; ARRIVE guidelines recommended for reporting animal research, and Sex and Gender in Research

Laboratory animals	C. elegans rrf-3(pk1426);glp-4(bn2)
Wild animals	Not relevant.
Reporting on sex	Not relevant.
Field-collected samples	Not relevant.
Ethics oversight	Experiments conducted on nematodes do not require institutional ethical clearance.

Note that full information on the approval of the study protocol must also be provided in the manuscript.
